# NMR Provides Unique Insight into the Functional Dynamics and Interactions of Intrinsically Disordered Proteins

**DOI:** 10.1021/acs.chemrev.1c01023

**Published:** 2022-04-21

**Authors:** Aldo R. Camacho-Zarco, Vincent Schnapka, Serafima Guseva, Anton Abyzov, Wiktor Adamski, Sigrid Milles, Malene Ringkjøbing Jensen, Lukas Zidek, Nicola Salvi, Martin Blackledge

**Affiliations:** † Université Grenoble Alpes, CEA, CNRS, IBS, 38000 Grenoble, France; ‡ National Centre for Biomolecular Research, Faculty of Science, Masaryk University, Kamenice 5, 82500 Brno, Czech Republic; § Central European Institute of Technology, Masaryk University, Kamenice 5, 82500 Brno, Czech Republic

## Abstract

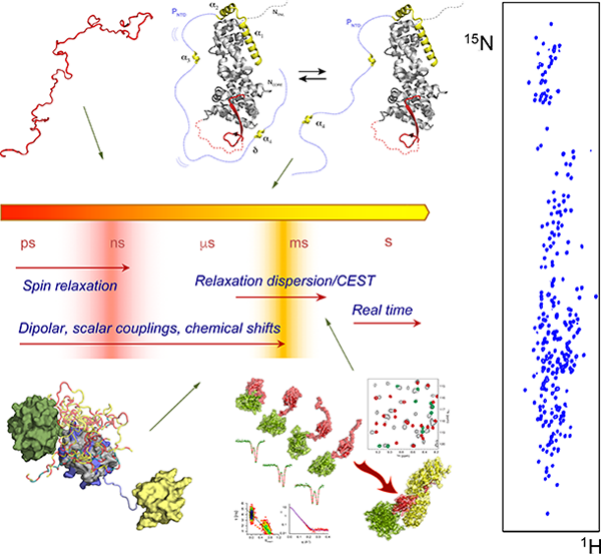

Intrinsically disordered proteins are ubiquitous throughout all known proteomes, playing essential roles in all aspects of cellular and extracellular biochemistry. To understand their function, it is necessary to determine their structural and dynamic behavior and to describe the physical chemistry of their interaction trajectories. Nuclear magnetic resonance is perfectly adapted to this task, providing ensemble averaged structural and dynamic parameters that report on each assigned resonance in the molecule, unveiling otherwise inaccessible insight into the reaction kinetics and thermodynamics that are essential for function. In this review, we describe recent applications of NMR-based approaches to understanding the conformational energy landscape, the nature and time scales of local and long-range dynamics and how they depend on the environment, even in the cell. Finally, we illustrate the ability of NMR to uncover the mechanistic basis of functional disordered molecular assemblies that are important for human health.

## 1 Introduction

Unexpected discoveries regularly revolutionize our understanding of molecular biology. The remarkable observation that intrinsically disordered proteins are prevalent throughout all known proteomes represents one such example, forcing a reassessment of established approaches for investigating biological function at the molecular level.^1−5^ Unlike folded proteins, the primary amino acid sequence of intrinsically disordered proteins (IDPs) does not adopt a stable tertiary fold to function but dynamically samples a broad free-energy surface. IDPs thus access a vast conformational landscape that nevertheless encodes specific biological activity.^6^ This conformational heterogeneity endows IDPs with considerable advantages over their folded counterparts, for example, the ability to interact with multiple partners, possibly simultaneously as in the case of hub-proteins. Combining transient and local disorder-to-order transitions with rapid dissociation rates allows efficient processing and provides the necessary level of multivalent, weak intermolecular binding to transiently form membraneless organelles^7^ (another phenomenon whose importance has revised our understanding of cell regulation and function). In general, although the potential benefits of conformational disorder are quite well discussed in the literature, we are still discovering the true breadth of functional diversity encoded in IDPs.

**Figure 1:**
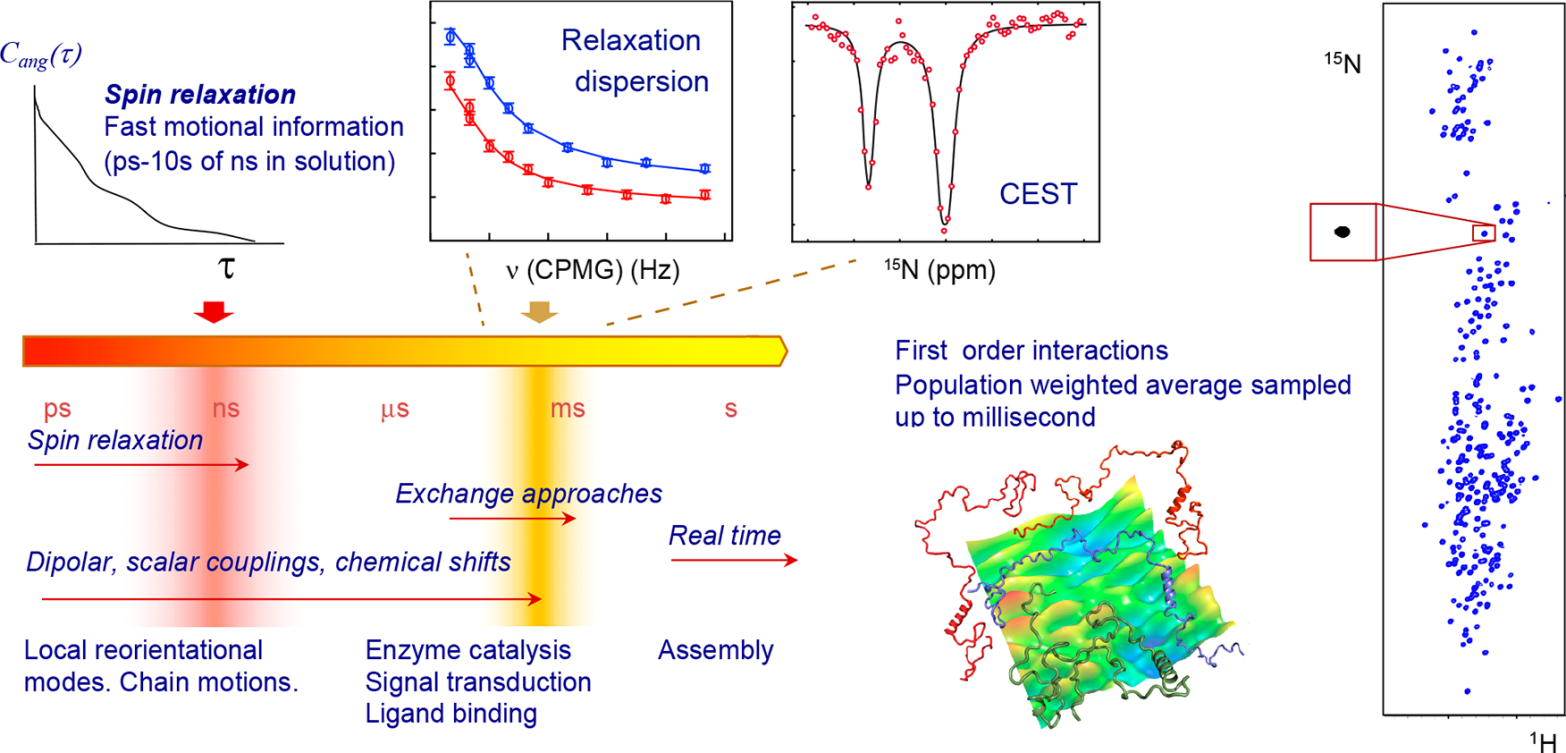
NMR probes biomolecular conformational changes on a vast range of time scales. NMR spin relaxation provides accurate information on the reorientational properties of relaxation-active interactions, normally interatomic bonds, up to tens of nanoseconds. In the fast exchange limit, a single NMR peak represents a population weighted average over the chemical shifts of each populated substate. When the exchange rate is in the same range as the difference in chemical shifts of the distinct states, on time scales from tens of microseconds to hundreds of milliseconds in proteins, line-broadening is observed, and ^1^H, ^13^C, and ^15^N NMR exchange approaches can be used to characterize interconversion between the different conformational states. Exchange that is significantly slower than the difference in chemical shifts of the distinct states gives rise to slow exchange, allowing all states to be individually investigated.

Structural dynamics are of course essential to biological function in all proteins, and the characterization of the conformational fluctuations that enable function is a vital aspect of our quest for a molecular understanding of biology. Complementary to the stabilization of distinct conformational substates and the determination of their three-dimensional structures at given points in a functional cycle, direct physical methods such as infrared,^8,9^ terahertz,^10^ neutron,^11^ dielectric^12^ Mössbauer,^13^ and Raman^14^ spectroscopies can be used to describe the characteristic time scales of protein motions. Time-resolved X-ray diffraction techniques^15^ and X-ray free electron lasers^16^ also provide simultaneous access to both high resolution structure and dynamics. Within the broad panoply of physical techniques available to characterize biomolecular dynamics, nuclear magnetic resonance (NMR) spectroscopy occupies a unique place, providing atomic resolution information over an incredibly broad range of motional time scales extending from tens of picoseconds to hours or even days (Figure 1).

Flexibility and dynamics not only define the physical nature but also the biological function of IDPs, and the two major challenges facing interpretation of experimental data from IDPs are related to these characteristics. The first concerns the accurate description of the conformational space sampled by the protein. NMR reports on a population-weighted average over the ensemble of interconverting states sampled at equilibrium so that as long as the exchange rates are fast on an NMR time scale, conformation-dependent parameters, such as chemical shift or scalar and dipolar couplings, report on interconversion between a potentially immense number of conformers. In practice for NMR studies of proteins using ^1^H, ^15^N, and ^13^C nuclei, this means interconversion on time scales faster than hundreds of microseconds. Interpretation of experimental data therefore requires statistical mechanical approaches to evaluate the nature of the conformational ensemble. The available degrees of conformational freedom that are accessible to IDPs significantly outweigh the ability of the experimental constraints to uniquely define the free-energy surface. Regardless of the approach used to delineate the conformational space, caution must therefore be employed to derive meaningful ensemble models that correctly describe the long-range and local conformational sampling. To this end, there has been considerable methodological development aiming to delineate the contours and limits of local and long-range conformational space sampled by IDPs in solution,^17−27^ from NMR, and other complementary biophysical techniques such as small angle scattering and single molecule Förster resonance energy transfer (smFRET).^28−32^ Progress in this direction has focused on the use of extensive exploration of conformational space, using for example stochastic sampling of the available degrees of freedom, and subsequent identification of combinations of conformers that when assembled into representative ensembles agree with experimental data and can describe the contours of the Boltzmann ensemble.^33−37^ The success of such approaches is predicated on the ability to accurately calculate the expected value of experimental data for a given conformation or conformational sampling regime. The same end can be achieved via ensemble restrained molecular dynamics simulation,^38−41^ for example, by including experimental data into the force field via a target function applied over the entire ensemble.^42−51^ The amount of detail concerning the conformational sampling of IDPs in solution that can be derived from all of these ensemble approaches depends of course heavily on the extent of experimental data available.^52^

The advantage of the fast exchange regime, reporting on a population-weighted average over an ensemble of states that interconvert on time scale faster than 100 μs, also highlights its key limitation that more precise information about the associated motional time scales is not explicitly contained in this average. Knowledge of the time scales of diffusion and chain dynamics, of interconversion rates between locally structured binding-competent and incompetent substates, and of transient contacts relating the conformational properties of distant regions of IDPs will all play an essential role in developing a deeper understanding of IDP reaction kinetics and thermodynamics. Understanding the dynamic properties of IDPs complements Cartesian descriptions of their exploration of conformational space, providing a new and essential dimension to our description of their functional behavior. In response to this challenge, time scales of conformational rearrangements of IDPs have been investigated using a vast range of experimental techniques,^53^ sensitive to local conformational dynamics such as infrared,^54,55^ Raman,^56^ or neutron spectroscopy^57−59^ or to long-range interactions using single molecule fluorescence,^60−69^ electron paramagnetic resonance,^70−72^ and NMR paramagnetic relaxation spectroscopies,^73−79^ but by far the most powerful technique is the use of NMR spin relaxation.

NMR spin relaxation probes the angular correlation functions of relaxation active mechanisms, typically dipole–dipole interactions between neighboring nuclei, arising due to reorientation processes of macromolecules on time scales ranging from 10s of picoseconds to 10s of nanoseconds or even slower. These time scales are also readily accessible to atomistic molecular dynamics (MD) simulation of fully solvated proteins, rendering the combination of MD and NMR extremely powerful. Advances in molecular simulation, in terms of accuracy of force-fields or sampling of slower dynamic time scales,^80−85^ have always accompanied advances in our understanding of the interpretation of NMR relaxation in terms of global and local molecular motions, demonstrating the synergy between these two atomic resolution techniques. Indeed, ^15^N and ^13^C NMR relaxation data have often been used to test and benchmark MD force fields and algorithms,^82,86−91^ establishing the accuracy of dynamic trajectories of soluble, folded proteins.

^15^N spin relaxation provides a remarkably sensitive probe of the motional time scales exhibited by IDPs, characterizing the dynamic properties of bond vectors throughout the length of the unfolded protein.^92^ The physical interpretation of the dynamic time scales contributing to the quenching of the angular correlation function is however less straightforward than in the case of folded proteins. The amount of information that can be extracted from spin relaxation is also limited by the efficiency with which fast large-scale motions quench the angular correlation function. ^15^N spin relaxation measurements in unfolded proteins have nevertheless been measured extensively, leading to the detection of extensive pico- and nanosecond motions, as well as clear correlations between motional time scales and structural propensities detected from chemical shifts and scalar and dipolar couplings.^93−114^

Further insight into the actual physical origin of the motional modes and time scales giving rise to NMR spin relaxation can again be derived from the combination of MD simulation with spin relaxation measurements.^115−118^ Measured relaxation rates report on population-weighted averages so that accurate simulation should account for fast motions occurring over the ensemble of states sampled by the protein. The value of relaxation rates associated with each substate depends on the nature of this conformation, so that in principle it would be necessary to simulate each of the substates and average the individual rates as a function of their populations, or to simulate sufficiently long trajectories to sample all individual states. In the case of globular proteins, the identification and simulation of distinct conformational substates that are in fast exchange on the chemical shift time scale but that exhibit distinct fast reorientational properties have indeed been shown to significantly improve the description of the ensemble of fast motions, as measured by the reproduction of experimental ^15^N relaxation rates.^119^ This demonstrates the improved accuracy of dynamic information when considering the entire free-energy surface but also the interdependence of fast and slower motions in proteins. For IDPs, this potential interdependence has an even greater importance and underlines the relevance of adequate sampling of the ensemble of conformational states.^120^

Despite major progress in the simulation of highly flexible or unfolded proteins,^42,118,121−123^ a more general application of these techniques has been hindered by the inability of state-of-the-art force fields to describe the dynamics of IDPs with acceptable accuracy.^90,124,125^ While the degrees of conformational freedom available to internuclear covalent bonds present in folded proteins are mainly dictated by the immediate environment, and therefore intraprotein interactions, for IDPs the solvent protein interactions take on a far greater importance, so that an imbalance between potential energy terms reporting on protein–protein and protein–solvent interactions^124^ may result in inaccurate kinetic and thermodynamic behavior. The resolution of this question, and the development of force fields that can describe both folded and unfolded proteins with equal accuracy,^126^ remains an important challenge.^90,120,124,127−130^ The availability of accurate and calibrated NMR relaxation rates from proteins with well-described conformational behavior will undoubtedly contribute to this important task.

Beyond the fast exchange regime, NMR relaxation experiments no longer represent a population-weighted average of the reorientational properties of the exchanging species but report on motions occurring on time scales defined by the difference in chemical shifts of the exchanging subspecies, in the range of micro to milliseconds. In this regime, NMR exchange spectroscopy is particularly powerful way to probe the molecular mechanisms underlying the exchange contributions, providing information on the thermodynamics, free-energy landscape, and kinetics of the interconversion between the species.^131−136^

Finally, our understanding of the functional modes adopted by IDPs is enriched by every physiologically relevant complex that is characterized experimentally. The functional interactome of IDPs is vast and potentially highly diverse, and our experimental sampling of the interaction modes employed by IDPs remains extremely punctual. Although specific model systems that are experimentally well-characterized provide useful bench-marks, insight into the true diversity of the IDP interactome requires more sampling, of more diverse systems, at atomic resolution. Exchange NMR, whether fast, intermediate, or slow, provides powerful tools to deliver this essential insight.

The aim of this review is to describe recent developments of NMR-based approaches to understand the conformational dynamic behavior of IDPs in physiological, and even cellular environments, and to illustrate the insight that NMR offers to reveal the mechanistic basis of functional disordered assemblies that are important for human health. Part of the power of NMR spectroscopy lies in the use of combinatorial approaches with structural techniques such as cryoEM and X-ray diffraction that provide the structural context within which the functional role of IDRs can be best understood. Examples will also be shown of the ability of NMR to characterize large-scale dynamics of complex biomolecular assemblies comprising highly disordered elements.

## 2 Accurate Mapping of the Conformational Landscape of IDPs

An accurate understanding of the conformational properties of IDPs, and intrinsically disordered regions (IDRs) of multidomain proteins, is of primordial importance. The dynamic behavior of IDPs is defined by the amino acid sequence, and the ability of the protein to interact via, for example linear motifs, is encoded and controlled by the intrinsic conformational sampling. In addition, IDRs, often linking folded domains, define the free-energy landscape of the protein, providing the degrees of conformational freedom of the entire molecular assembly.^6,137−139^ Characteristics such as charge and hydrophobicity distribution of IDPs have been interpreted in terms of their role in controlling physical parameters, for example, compactness and extendedness,^140^ and the ability of IDPs to participate in multivalent interactions.^141−144^ Similarly, regulation of these degrees of freedom can be achieved by post-translationally modifying the chemical nature of the chain.^145−148^

Two recent studies described herein illustrate the importance of a detailed consideration of the averaging properties of different experimental data types to understand the conformational nature of IDRs. In particular, the combination of long-range and local transient structure poses specific challenges to the analysis of disordered proteins in terms of representative ensembles, and certain pitfalls must be avoided to extract accurate structural information.

Chemical shifts and scalar couplings present two important features that directly impact their interpretation. First, they depend primarily on the local structural environment of the observed spin, and second, if interconversion between the different states is much faster than the difference between the expectation values of the different states in isolation, the measured NMR spectrum represents a weighted average of the ensemble of states. Conversely, parameters whose experimental values depend on time-dependent interactions, such as paramagnetic relaxation for example, require a more detailed consideration of the averaging properties, as has been discussed.^79^ Residual dipolar couplings (RDCs) depend on the average of the orientations of the internuclear vector (*I*–*S*) with respect to the magnetic field,
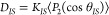
1where *K*_*IS*_ describes physical constants such as the gyromagnetic ratio and the internuclear distance, and *P*_2_ (*x*) = (3*x*^2^ – 1)/2. In a molecule of fixed shape, we can expand this average,
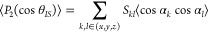
2where *α*_*k*_ refers to the orientation of the internuclear vector with respect to a traceless second rank tensor **S** that describes the alignment properties of the molecule.

In highly flexible proteins, **S** can clearly vary significantly over the ensemble such that proteins of different shape, and therefore different alignment properties, but identical local sampling, would give rise to very different RDCs:
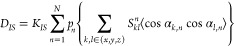
3Using simple and intuitive simulation of target ensembles, it was demonstrated that ensemble descriptions derived from RDCs of molecular systems whose shape varies significantly over the ensemble can actually reproduce experimental data very closely, even without explicit consideration of the alignment properties of the component conformations. However, the orientational properties of the internuclear vectors are then severely compromised and inaccurately describe the conformational space compared to the target ensemble.^149^ This reiterates the long-held observation that to accurately describe local and long-range conformational sampling, it is necessary to respect both of these contributions to the average over the ensemble of states.^150^

The importance of considering long-range order in the interpretation of RDCs was also illustrated in a recent study of the δ domain of RNA polymerase (δ−RNAP), where multiple NMR parameters and small angle scattering data were combined using the ensemble selection approach, ASTEROIDS, to compare the free energy landscape of different forms of the protein. ASTEROIDS uses extensive conformational sampling described in an initial prior database, broadly sampling amino-acid specific statistical-coil distribution for the unfolded chain,^151,152^ and a genetic algorithm, to select representative subensembles of conformers that in combination are in agreement with the experimental data. The sampling of the prior database is modified iteratively until convergence is achieved within the estimated uncertainty.^37^

In the case of δ−RNAP, the 90 amino acid C-terminal IDR follows the similarly sized folded domain.^153^ The IDR is locally highly charged, with mainly acidic but also basic stretches of amino acids. As in the case of a number of acidic disordered domains in RNA-polymerase machinery, the acidic sequence has been suggested as an RNA mimic.^154^

**Figure 2:**
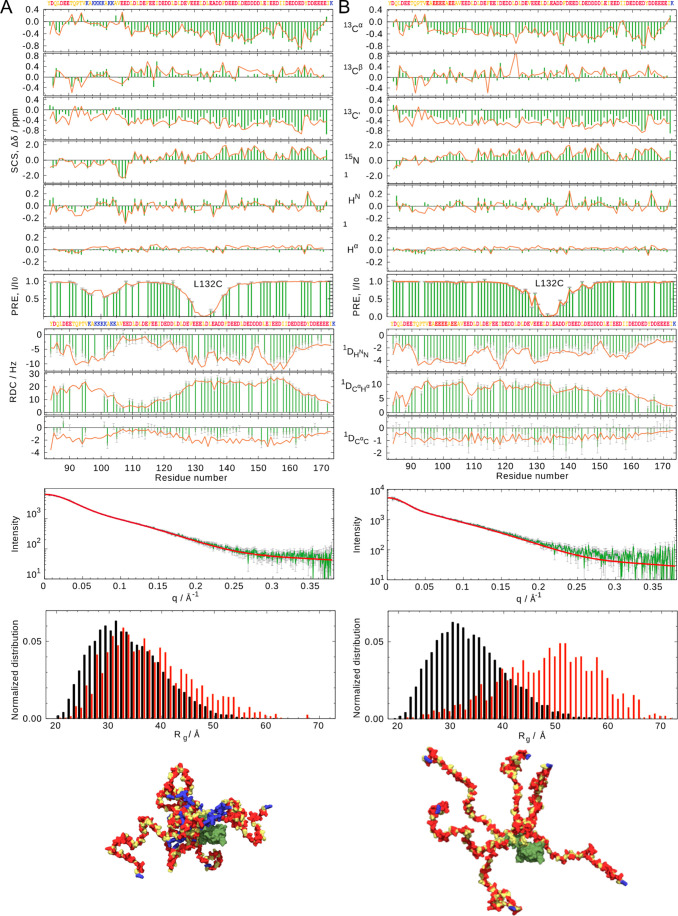
Experimental comparison of conformational behavior of the intrinsically disordered δ subunit of bacterial RNA polymerase. (A) Experimental parameters measured on wild-type protein (green bars) compared to ensemble-averaged values calculated from 10 ensembles comprising 200-strong ASTEROIDS ensembles (red lines). From top to bottom: secondary chemical shifts, paramagnetic relaxation enhancements (labeled at residue 132), residual dipolar couplings, and SAXS. Bottom: comparison of distribution of radii of gyration from a statistical coil pool (black) and the ASTEROIDS ensemble (red). Structural models of five conformations are displayed below the plots (ordered domain in green, IDR in yellow with positively and negatively charged residues highlighted in blue and red, respectively. (B) Same parameters for the mutated protein in which a lysine-rich tract ^96^KAKKKKAKK^104^ are replaced by ^96^EAEEEEAEE^104^. This results in a clear abrogation of long-range contacts with the C-terminal half of the domain that collapse the protein. This collapse, and its abrogation, are visible not only in SAXS and PRE data but also in the residual dipolar coupling data. (Reproduced with permission from Kuban et al. 2019 Copyright 2019 ACS^156^).

Experimental data used to describe the conformational sampling of the IDR included ^13^C, ^15^N, and ^1^H backbone chemical shifts, paramagnetic relaxation enhancements (PREs), residual dipolar couplings (RDCs), and small-angle X-ray scattering data. PREs provide clear evidence of transient long-range order in the IDP, with apparent contacts between regions exhibiting opposite charges (Figure 2).^155^ Analysis of δ-RNAP in terms of representative ensembles results in close agreement with expected behavior of the averaged RDCs. Characteristic modulations of multiple RDCs were observed in each peptide unit (manifest as quenching of the RDCs measured between the points of contact), and these RDCs were only correctly predicted when the long-range contact identified from the PREs was included in the analysis.

Mutation of the cluster of basic amino acids to acidic residues abrogates the long-range contacts, resulting in extinction of the characteristic PRE- and SAXS-derived evidence of compaction in the wild type protein, revealing a highly extended IDR in the absence of the basic cluster, and a disappearance of the characteristic long-range RDC modulation. The combined analysis thus results in an accurate, integrated description of the ensemble of states sampled by both wild-type and mutant protein in solution, providing insight into the impact of the electrostatic charge distribution on local and long-range conformational behavior.^156^ Interestingly, the loss of long-range contacts induced by mutagenesis influences cell fitness and transcription efficiency *in vitro*. While the complete knockout of the delta subunit makes transcription too fast and insensitive to regulation by initiating nucleoside triphosphates, the mutation disrupting long-range contacts has the opposite effect: it inhibits transcription from promoters that form unstable complexes with RNA polymerase.

## 3 NMR Studies of IDP Dynamic Modes and Timescales

### 3.1 NMR Relaxation of IDPs and Models of Correlation Functions

As introduced earlier, NMR relaxation occurs due to angular fluctuations of relaxation-active interactions resulting in transitions and incoherent dephasing that relax the spin state back to equilibrium.^92,157,158^ The angular reorientation of such interactions can be described in the time domain (correlation function *C*(*τ*)) or the frequency domain (the spectral density function *J*(*ω*)). Protein backbone dynamics are typically characterized in solution using longitudinal (*R*_*1*_) and transverse (*R*_*2*_) autocorrelated ^15^N relaxation rates, heteronuclear ^1^H–^15^N cross-relaxation, and ^15^N longitudinal (*η*_*z*_) and transverse (*η*_*xy*_) cross-correlated dipole–dipole/CSA (chemical shift anisotropy) cross-relaxation (*σ*_*NH*_).^92^ The advantage of measuring different rates lies in their distinct dependence on different combinations of the angular spectral density function at the characteristic Larmor frequencies defined by the spin system, ω_N_, ω_H_, ω_H_ ± ω_N_.

If enough measurements are available, the spectral density functions can be mapped from the different relaxation rates^159,160^ using reduced spectral density mapping^161−164^ to estimate *J*(0), *J*(ω_N_) and an approximate mean value at high frequencies <*J*(0.87ω_H_)> throughout the sequence. Alternatively, the correlation function of internal motional modes can be described analytically, in terms of geometric and temporal parameters (for example, n-site jumps of diffusion in a cone), although it can be difficult to differentiate between these models on the basis of NMR relaxation rates alone. A simple and popular alternative is to use the model-free approach, where mathematical contributions to the autocorrelation function are parametrized. The approach is simply understood in the case of internal modes in a folded protein,^165−169^ where it is possible to express the angular correlation function as
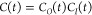
4where *C*_*O*_ (*t*) is the correlation function for global motion, and a faster internal contribution, that is not associated with a specific motional mode, describes restricted motion relative to the molecular frame:
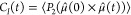
5where μ̂ is a unit orientation vector of the relevant relaxation-active interaction (dipolar or CSA).

If the internal correlation function *C*_*I*_(*t*) is approximated to a single exponential, the associated spectral density function can be described as
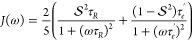
6where τ_*e*_^’^ = (τ_*R*_^-1^ + τ_*e*_^-1^)^−1^*τ*_*R*_ describes the overall rotational diffusion and S^2^ is the generalized order parameter. Extension^168^ to two internal components with distinct correlation times (*τ*_*f*_ and *τ*_*s*_ and order parameters, gives)
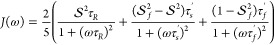
7where τ_*s*_^′^ = (τ_*R*_^–1^ + τ_*s*_^–1^)^−1^, τ_*f*_^′^ = (τ_*R*_^–1^ + τ_*f*_^–1^)^−1^.

This formalism is commonly used to interpret relaxation measured in folded proteins, with the global contribution to the autocorrelation and spectral density functions assumed to be common for all sites. Although, due to their high flexibility, IDPs are not expected to exhibit a shared diffusion tensor for distinct regions in the chain, the same mathematical formalism can be used to model the spectral density functions of each site independently, assuming that the time scales of the component modes are sufficiently separated, and that all the motions are isotropic:
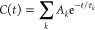
8with ∑_*k*_*A*_*k*_ = 1, and
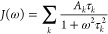
9

This formalism has been diversely exploited for the interpretation of relaxation from partially denatured proteins and IDPs.^93,95,97,170−173^ Alternatively, it is possible to describe the spectral density function in terms of an analytical distribution of motions, of which the model-free approach represents one of the simplest manifestations.^99,103,110,174^ Here again, the complexity of the models makes differentiation difficult, although they have been successfully used to explain the dynamic behavior of synthetic homopolymers,^175^ and surely provide a more physical representation of the complex dynamics of flexible proteins.^103^

In highly dynamic molecules such as IDPs, large-amplitude motions occur in the range of nanoseconds,^93−114^ rapidly quenching angular correlation and reducing the slowest sensitive time scales to the nanosecond range (at room temperature and in free solution). Nevertheless, the existence of segmental motions was suggested from the bell-shaped dependence of transverse relaxation components (with respect to primary sequence, tailing off to low values at both termini), in chemically denatured and intrinsically disordered proteins,^104^ relating to stiffness or side chain bulkiness,^96,176^ and from ^1^H relaxometry.^177^ IDRs connected to folded domains have been shown to induce slower components on the rotational diffusion properties of multidomain proteins indicating the importance of local viscosity and drag on dynamic time scales.^178−181^ Faster time scales are expected to relate to more local dynamics, for example, of backbone dihedral angles, which may be important in terms of local folding or binding;^6,23,182−192^ however, in general the physical origin of observed relaxation rates remains weakly characterized.

### 3.2 Recent Applications of Model-Free Approaches to IDPs

It is clear from eq 9 that amplitudes and time scales of the different components may be correlated and that the resulting parametrization will depend on the accurate estimation of the number of contributions. In the context of identifying the most appropriate model for the accurate interpretation of NMR relaxation from IDPs, a number of recent studies used extensive data sets to shed important light on the available information content. Rather than fixing the number of models and determine the most appropriate correlation times, Ferrage and co-workers^193^ used an array of fixed correlation times (*τ*_*k*_), distributed on a logarithmic scale, with variable amplitudes (*A*_*k*_), that could also be zero, to analyze the spectral density function from eq 9. The backbone dynamics of the partially disordered protein Engrailed 2 were analyzed using a large range of auto- and cross-correlated relaxation rates measured at five magnetic fields between 400 and 1000 MHz ^1^H frequencies. This provides a grid of motional amplitudes corresponding to six characteristic correlation times for the entire protein, clearly delineating the folded and unfolded domains, and revealing dominant time scales around 1 ns in the unfolded domain.

Gill et al.^194^ also studied the dynamics of a partly unfolded protein, the basic leucine-zipper region of GCN4. In this case, ^15^N *R*_1_, *R*_2_, and *σ*_*NH*_, measured at 600, 700, 800, and 900 MHz ^1^H frequency were analyzed by rearranging the measured relaxation rates using a modified spectral density mapping, and comparing these results to a model free analysis using eq 9 to determine how many independent contributions can be extracted from this analysis. The results demonstrate that the extended model-free approach accurately describes the experimental data as well as being statistically justified on the basis of the experimental uncertainty. The authors note that more than three contributions cannot be theoretically justified from these data.

A similar study of the dynamic behavior of the 126 amino acid C-terminal disordered domain of Sendai virus nucleoprotein (NT), examined ^15^N *R*_1_, *R*_2_, *σ*_*NH*_, *η*_*z*_, and *η*_*xy*_ measured at four magnetic field strengths (600, 700, 850, and 950 MHz ^1^H frequency). In a first step, autocorrelated and cross-correlated rates measured at each field strength were analyzed using reduced spectral density mapping at each magnetic field strength, confirming the self-consistency of the data, and the absence of exchange contributions to *R*_*2*_. The data were then analyzed using eq 9 to determine the optimal number of contributions. Two procedures were undertaken, the first based on statistical testing, to determine the minimum number of contributions for each site. Models with 2 (τ _1_ and θ), 4 (τ _1_, τ_2_, *A*_*2*_, and θ), 5 (*A*_2_, *A*_3_, τ _2_, τ_3_, and θ), or 6 (*A*_2_, *A*_3_, τ _1_, τ _2_, τ_3_, and θ) parameters for all sites in the molecule, corresponding to 1, 2, or 3 contributions to the relaxation-active correlation function. The 3-component model was found to be justified throughout the protein. Second, 10% of all data were removed from each data set, and their values predicted from the parameters determined from the remaining data sets, again demonstrating that 3 components are essential to correctly predict experimental values. This implies that sufficient relaxation data have been measured to justify the more complex model.

Experimentally measured relaxation rates vary significantly throughout the length of IDPs, exhibiting apparent correlation with transient secondary structure/linear motifs and differential dynamic behavior depending on sequence composition. It is therefore interesting to investigate the physical origin of the three components. The ability to measure NMR relaxation rates in complex environments such as liquid–liquid phase separation^195−197^ and *in cellulo*(198) also calls for a careful analysis of the possible physical mechanisms underlying these experimentally observed dynamic modes. To this end, two approaches, described below, have recently shed more light on the information content of this site-specific variation of relaxation in IDPs, in particular concerning the relative importance of local backbone conformational sampling and long-range chain-like behavior. The first concerns the dependence of the different components on environmental parameters such as temperature and crowding, and the second combines novel MD-based approaches to the interpretation of relaxation in IDPs.

## 4 Developing a Unified Description of IDP Dynamics in Solution

### 4.1 Temperature-Dependent Relaxation Reveals Properties of Distinct Dynamic Modes

**Figure 3:**
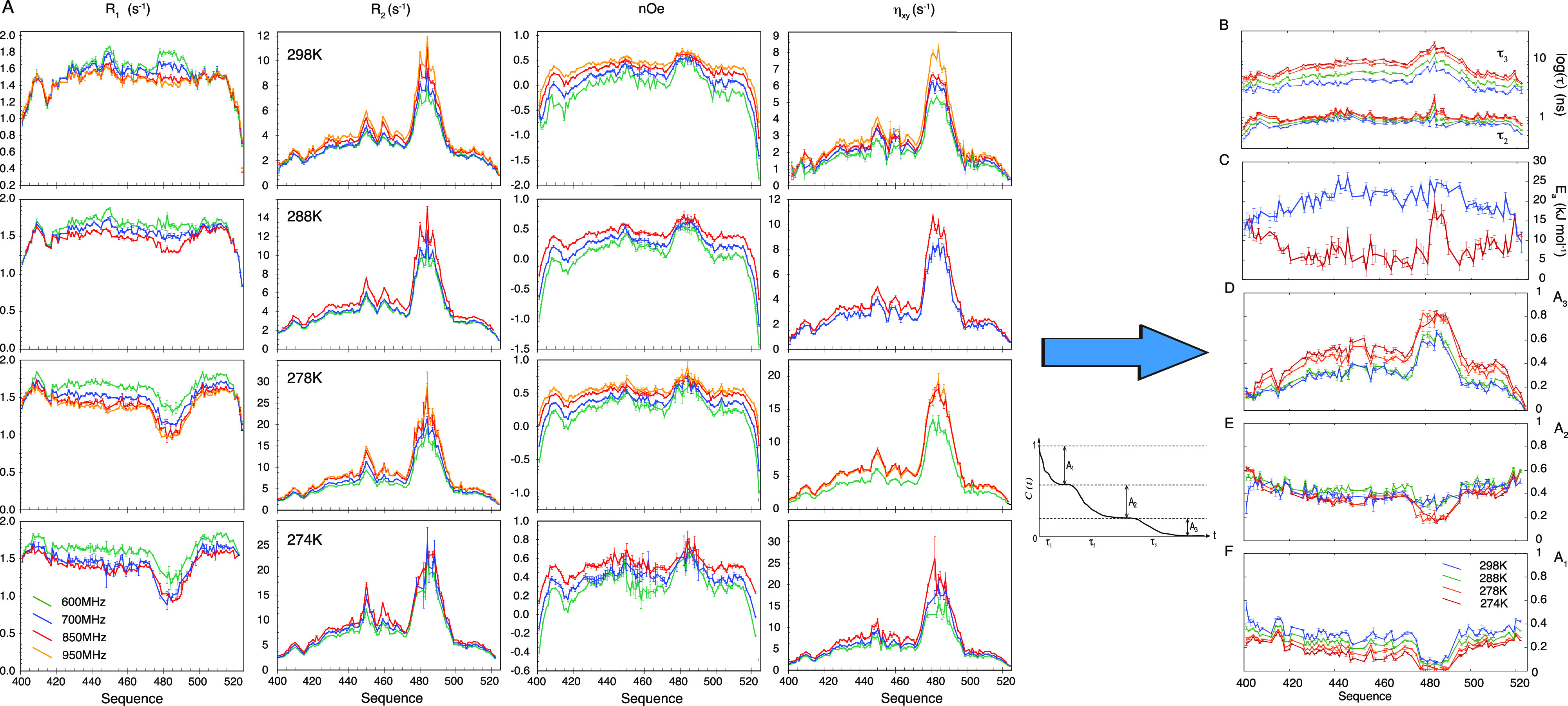
Temperature-dependent ^15^N relaxation maps three modes of intrinsically disordered protein dynamics. (A) ^15^N auto- and cross-relaxation rates of NT measured at different magnetic field strengths (green, 600 MHz ^1^H frequency; blue, 700 MHz; red, 850 MHz; orange, 950 MHz) and at different temperatures (top: 298 K, second row 288 K, third row 278 K, bottom 274 K). (B–F) Analysis of all relaxation data in (A), using a three-component model-free approach, with characteristic correlation times related via an Arrhenius expression. (B) Slow (τ_3_) and intermediate (τ_2_) correlation times at 274 K (red), 278 K (orange), 288 K (green), and 298 K (blue). (C) Activation energies for slow (red) and intermediate (blue) time scales for each residue. (D–F) Amplitude of slow (D), intermediate (E), and fast (F) time scale contributions (Reproduced with permission from Abyzov et al. JACS 2016 Copyright 2016 ACS^199^).

The study of NT, a disordered protein containing a short helical linear motif was extended to measure *R*_1_, *R*_2_ and *σ*_*NH*_, and *η*_*xy*_ and *η*_*z*_ at four magnetic field strengths (600, 700, 850, and 950 MHz) and over a large range of temperatures (268–298 K) (Figure 3A).^199^ Up to 61 rates were measured for each amide group in the protein and interpreted using a simple Arrhenius relationship to couple the correlation times at the different temperatures (in analogy to the study of the temperature-dependent response of a microcrystalline protein by solid state NMR^200^):
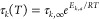
10

The different temperature dependences of the three components are described by temperature coefficients, or activation energies given by *E*_*k,a*_ (*τ*_*k,∞*_ is the Arrhenius prefactor). Fitting to this function requires the determination of parameters defining the relative amplitude of the three components at each temperature and the effective temperature coefficients of the intermediate and slowest contribution (the fastest contribution around 50 ps shows insignificant temperature dependence). Again, cross-validation by removal of either 10% of all data, or data from each magnetic field, indicates that the analysis is satisfactorily overdetermined. It is worth pointing out that predictive cross-validation is not so common in analysis of protein dynamics from NMR spin-relaxation but when applied shows a reassuring level of confidence in the data analysis.^199^

The simultaneous analysis of data from all five temperatures (Figure 3B–F) reveals fascinating insight into the origin of the three resolved components. The amplitude of the slowest component exhibits a bell-shaped distribution with respect to primary sequence, with a clear maximum in the helical region. The time scale parallels this distribution, reaching time scales up to 25 ns in the helical region at 268 K. Although this contribution is dominated by the slowest times experienced by the helix, the effective activation energy, or rate of change of *τ*_*3*_ with temperature, exhibits a smooth function along the sequence, reaching a maximum (20–25 kJ mol^–1^) in the center of the sequence. It was proposed that the slowest contribution reports on chain or segmental dynamics. The reason that slower motions are detected in the helix is that *C*(*t*) is not as efficiently quenched by the high amplitude fast motions occurring in the remaining unfolded part of the chain. The residual order left after the more restricted fast motions occurring in the helix allow for the detection of slower motion that has little effect on correlation functions from the less-structured parts of the chain. This is further supported by the analysis of data measured using protein constructs engineered to comprise 50, 75, or 126 amino acids, revealing a clear dependence of the *τ*_3_ on the length of the peptide chain, as expected for chain dynamics considered using Rouse or Zimm models.^201−203^

The intermediate motion has a much flatter distribution over the unfolded regions, and the apparent activation energies are in the range expected from studies of peptide backbone free energy landscapes.^204,205^ In this case, there is a discontinuity in activation energy between the unfolded and helical regions, motivating the suggestion that these contributions report respectively on local fluctuations within Ramachandran wells and constrained internal dynamics or partial unfolding in the helix.^54,128,206,120^

Although relaxation in IDPs is often thought to provide information essentially concerning subnanosecond motions, the analysis shown here clearly demonstrated that short, structured motifs in unfolded polymers are also dependent on slower, segmental or chain-like motions, or whatever other motion finally quenches the angular correlation function. Most regions are not sensitive to these motions because of the extent of the faster motions, but if one can locally quench these, a great deal of insight can be derived from the resulting relaxation rates.

We note that while the contribution of the slowest motion increases at lower temperatures, as the fastest motion falls, the amplitude of the intermediate motion systematically passes through a maximum at 288 K. This may provide us with information about the shape of the actual distribution of correlation times and their impact on the sampled correlation function.

### 4.2 IDP Dynamics under Crowded Conditions Experienced *In Cellulo*

Although significant progress has thus been made over recent years in our understanding of the information provided by NMR relaxation studies of IDPs, it remained unclear how to interpret data measured in more complex, and more specifically in the more crowded, physiological environments in which they function.^208,209^ This question is particularly relevant with respect to NMR *in cellulo*,^198,210−216^ where IDPs function in environments with molecular concentrations reaching 400 g/L,^217−219^ very likely strongly affecting the time scales of IDP dynamics.^220−222^ The effect of local environment on IDP function is also relevant for understanding the mechanistic role of IDPs in membraneless organelles.^195−197,223−225^ IDPs are subjected to extreme solvent accessibility compared to folded proteins, suggesting that the physiological environment in complex multicomponent environments will very likely strongly influence dynamic modes and time scales. Single molecule fluorescence techniques have provided unique insight into the importance of so-called internal and solvent friction on IDP dynamics and partially folded or destabilized protein states as well as on the kinetics of protein folding.^66,226,227^ These approaches have been used to investigate the dynamics of IDPs^228,229^ and protein function^230^ in the cellular environment.

Similarly, NMR spectroscopy has been used to investigate modulation of the folding/unfolding equilibrium of globular proteins *in cellulo*, indicating changes in both population and exchange rates as a function of the cellular milieu, and a dependence on weak, so-called quinary^231^ interactions between the protein of interest and diverse other molecules constituting the intracellular matrix.^232−234^ NMR was also used to describe the impact of the cellular milieu on protein dynamics, from small globular proteins to IDPs.^210,211,215,235−238^ In a detailed study, Theillet and co-workers compared the influence of different viscogens on the dynamics of α-synuclein, with ^15^N relaxation measurements made in mammalian cells, revealing changes in dynamics of the termini of the protein, presumably associated with crowding-induced compaction or inter- and intramolecular interactions. The extent of changes appeared to be more pronounced *in cellulo*, suggesting additional impact of intermolecular interactions on the relative deceleration of the NH-backbone fluctuations.^198^ In the context of these examples, and the growing body of experimental data,^239−244^ a physical framework that incorporates the effects of molecular crowding on the dynamics of the protein would provide a welcome tool allowing quantitative interpretation of NMR relaxation measured under physiological conditions.

**Figure 4:**
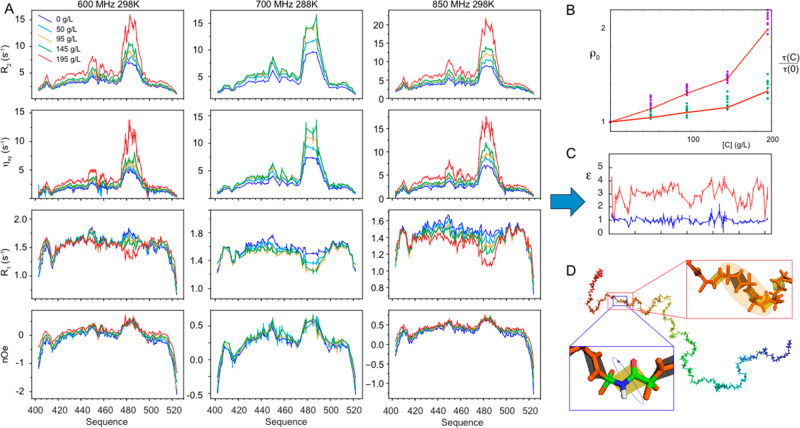
Viscosity-dependent ^15^N relaxation maps distinct response of local and longer-range dynamics in intrinsically disordered proteins. (A) Transverse (*R*_2_) and longitudinal (*R*_1_) relaxation, transverse cross-correlated DD/CSA (η_*xy*_) and heteronuclear {^1^H}-^15^N nuclear Overhauser enhancement (NOE) recorded at 600, 700, and 850 MHz as a function of concentration of Dextran 40. (B) Longitudinal water relaxation (solid red line, normalized to the value in free solution; ρ_0_) shows a similar dependence on concentration of viscogen to the intermediate time scale motion (green points). The slow motional component (purple) resembles approximately 3* ρ_0_ (dotted line). (C) Friction coefficients (ε) for intermediate backbone (blue) and slower, segmental (red) motions. (D) Cartoon representation of the length scales of intermediate and slower motions (Reproduced with permission from Adamski et al. JACS 2019^207^ Copyright 2019 ACS).

Recent work further addressed this challenge by measuring dynamics of IDPs as a function of environmental complexity. An extensive set of multifield NMR relaxation rates were measured over a broad range of conditions, using inert crowding agents to systematically modify viscosity, as well as temperature (Figure 4).^207^ This calibration allowed the dynamics of two IDPs to be mapped as a function of environmental conditions, including both viscosity and temperature. The two IDPs exhibit distinct physical properties, comprising both partially folded and highly flexible elements. Local, or nanoviscosity was gauged by measuring ^1^H longitudinal relaxation of water,^157^ which, at the high magnetic fields used here, is expected to be dominated by rotational diffusion of the water molecules.^245−247^ The overall dependences of the nanoviscosity of the solvent and solute on the concentration of viscogen show similar features, with the intermediate and slow correlation times of the backbone of the protein, and the ^1^H R_1_ both deviating from the linear regime in the range of 200 mg/mL (Figure 4). Nevertheless, the two motional modes of the protein backbone exhibit very different responses, with friction coefficients that are much steeper (approximately a factor of 3) for the slower motions. As noted from fluorescence-based studies, viscosity probes of different dimensions are expected to measure different effective viscosities,^248−252^ so that friction coefficients would be expected to be characterized by distinct length scales and to decrease for smaller probes.^253,254^ This suggests, perhaps not surprisingly, that intermediate and slow dynamic modes are associated with fragments of different dimensions, for example, respectively, single and multiple peptide units. The ratio of friction coefficients corresponding to intermediate and slow motions was reproduced for both experimental systems (over 200 amino acids), suggesting that the observation may be general. The observed differences in effective friction coefficients may be related to observations made by Schuler and co-workers that translational diffusion slows down considerably more than rotational diffusion of the IDP prothymosin α inside crowded cells, suggesting very different length scales and susceptibilities to crowding.^229^

**Figure 5:**
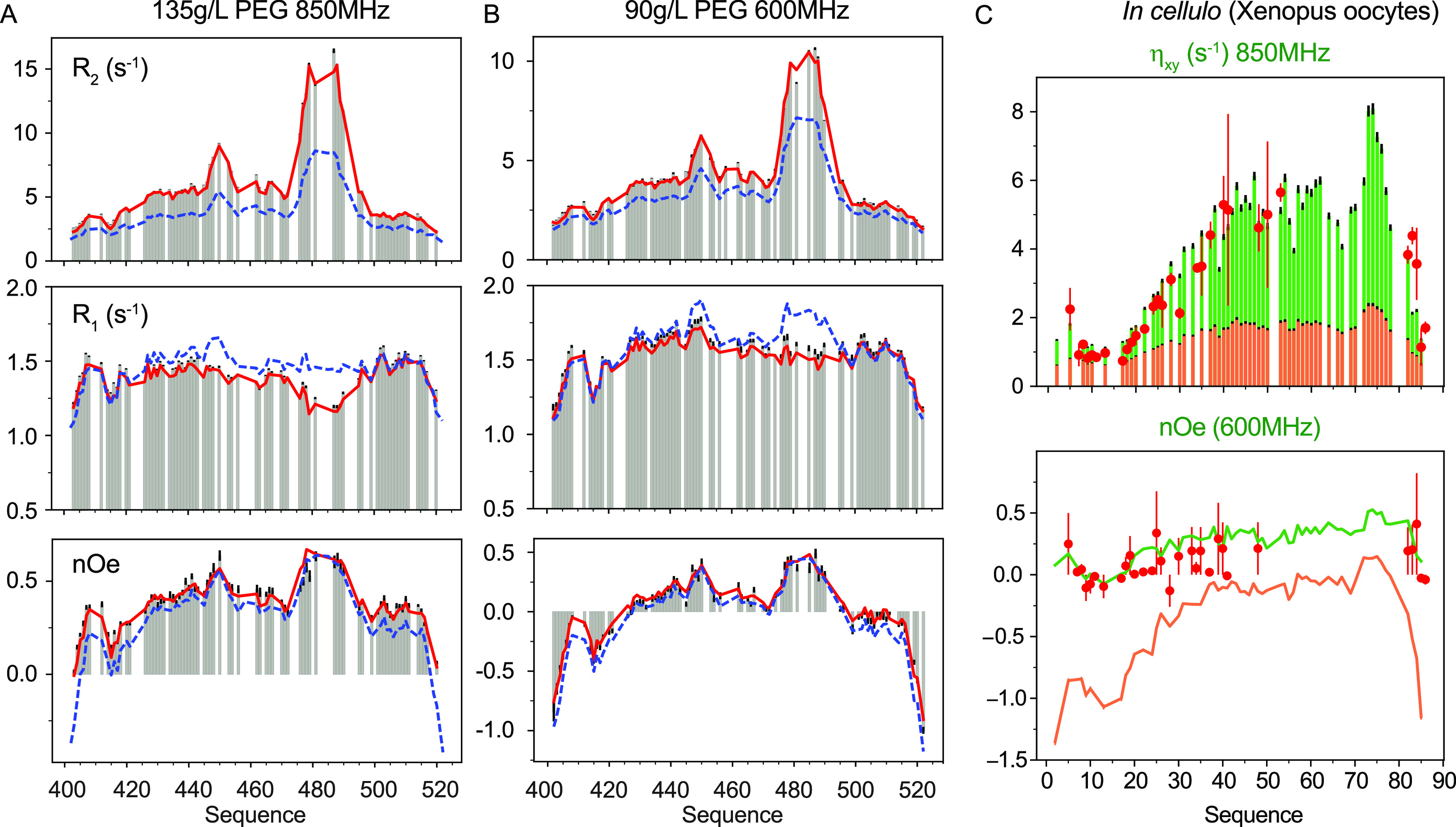
Residue-specific friction coefficients are transferable between different *in vitro* crowding environments and even predict values measured *in cellulo*. (A) Experimental ^15^N relaxation rates recorded on Sendai virus NT in the presence of 135g/L PEG (gray bars) compared to values calculated using sequence-specific friction coefficients (eq 11) (red lines) determined as a function of Dextran concentrations and water relaxation in the sample of interest. For comparison, relaxation rates predicted under dilute conditions are shown in blue. (B) Relaxation rates measured at 600 MHz ^1^H frequency at a concentration of 90 g/L PEG (colors as in (A)). (C) ^15^N relaxation rates recorded in-cell (red points) compared to values calculated on the basis of dynamic parameters determined *in vitro* (green bars and line). Orange bars and lines show rates predicted for dilute conditions. Experimentally determined friction coefficients and the experimental measurement of the water *R*_*1,0*_*in cellulo* were used in the prediction. (Reproduced with permission from Adamski et al. JACS 2019^207^ Copyright 2019 ACS).

On the basis of these observations, it was possible to develop, and test, a single expression to describe the dynamic modes and their characteristic time scales of IDPs in complex mixtures, their temperature and viscosity coefficients, using a minimal set of physical parameters to relate both the intermediate and slow time-scales (*τ*_*k*_) to the nanoviscosity of the solvent:
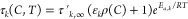
11where ρ(*C*) = (*η*_*C*_ – η_0_)/η_0_ = (*R*_1,*C*_ – *R*_1,0_)/*R*_1,0_, and *R*_1,0_ and η_0_ are the longitudinal relaxation rate of water and the viscosity in the absence of viscogen, *R*_1,C_ is the longitudinal relaxation rate, *η*_*C*_ is the viscosity, and *τ′*_*k*__,∞_ is a prefactor representing the correlation time at infinite dilution and temperature. *ε*_*k*_ is the residue-specific friction coefficient relative to *η*_*C*_ of intermediate or slow motions. The model turns out to be robust and remarkably transferable *in vitro*. For example, once sequence-specific friction coefficients have been determined as a function of concentration for a particular protein, highly sensitive dynamic probes such as a complete set of ^15^N relaxation rates measured in very different crowding conditions are predicted with very high accuracy, simply on the basis of the measurement of the water *R*_1_ (Figure 5A).

Perhaps most remarkably, the expression reproduces experimental relaxation measured *in cellulo* in *Xenopus* oocytes, on the basis of viscosity coefficients measured *in vitro* and nanoviscosity measured in the cell (Figure 5B). This unified description offers new insight into the nature of IDPs, and extends our ability to quantitatively investigate their conformational dynamics in complex environments. Such a successful application of experimental methodology from *in vitro* viscogen to *in cellulo* observation may appear surprising in view of the complexity of the cellular environment^255^ and the evident inability of synthetic polymers to reproduce this complexity.^256^ This study suggests that such concerns do not prevent the accurate prediction of average reorientational properties of IDPs in cells and indicates that the averaging of observable signals from IDPs and water remain closely coupled even in the multicompartmental environment of the cell.

## 5 Interpreting NMR Relaxation in IDPs Using MD Simulation

### 5.1 Accounting for Ensemble Conformational Sampling to Interpret Relaxation from IDPs

Although MD simulation provides unique insight into the conformational dynamics of IDPs,^42,118,122,123^ force-fields that accurately describe the behavior of folded proteins often fail to reproduce ensemble averaged properties of IDPs in solution, probably due to the importance of protein–solvent interactions. This in turn has motivated the conception of force fields that have been specifically designed for IDPs.^90,120,124,127−130^

**Figure 6:**
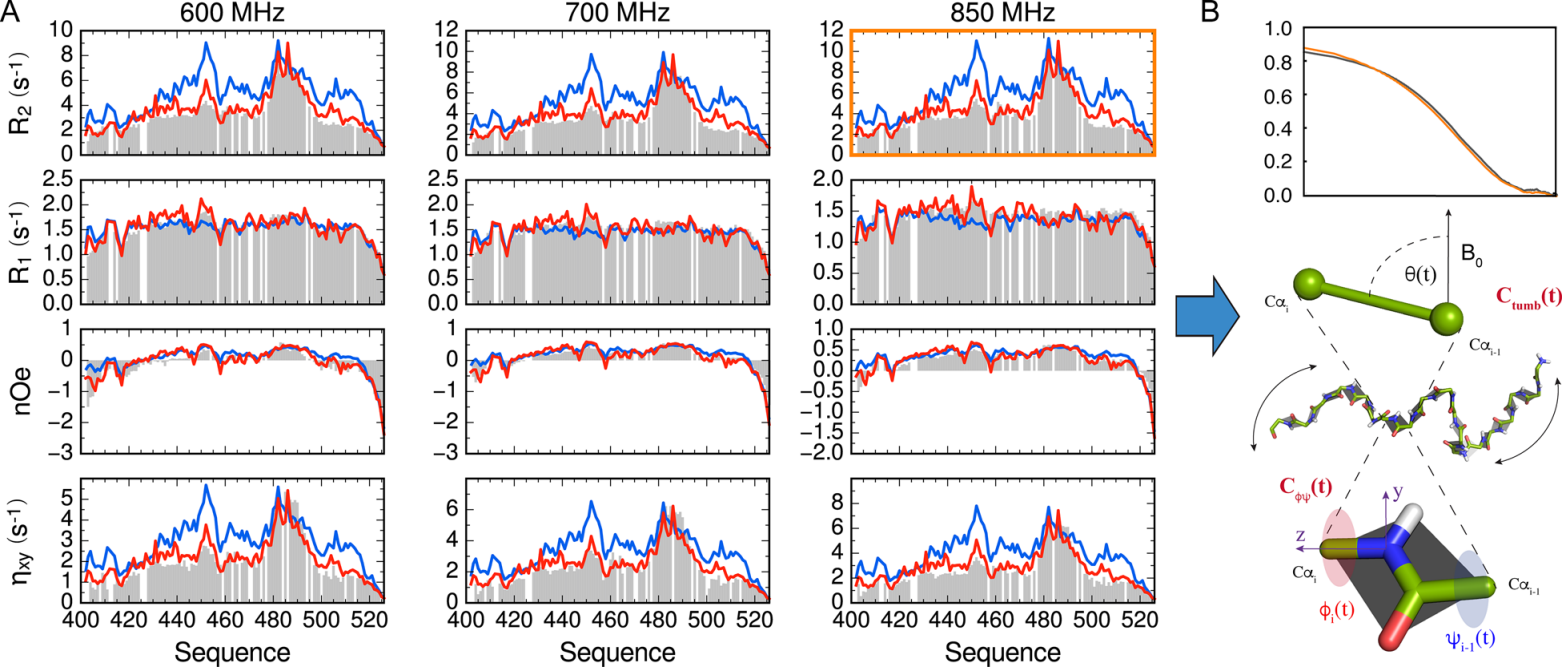
NMR relaxation allows for the identification of ensembles of time-dependent trajectories that represent fast motions in interconverting substates. (A) Experimental ^15^N relaxation rates recorded on Sendai virus NT at 298 K in dilute conditions (gray bars) compared to values calculated from 4 μs of MD simulation, (blue line). The red line shows values calculated from the ABSURD procedure targetting only transverse relaxation measured at 850 MHz (orange box). (B) The ABSURD procedure results in average time-dependent correlation functions that can be decomposed into local and segmental motions of the peptide chain. (Reproduced with permission from Salvi et al. JPCL 2016^125^ Copyright 2016 ACS and Salvi et al. Angewandte Chemie 2017^257^ Copyright Wiley 2017).

Spin relaxation remains the most powerful NMR observable to characterize dynamic time scales at a sequence specific level, and reproduction of experimental values is often the most challenging for MD simulation. As described earlier, assuming conformational exchange that is fast on the chemical shift (and relaxation rate) time scale, experimentally observed rates derive from a population-weighted average over individual relaxation occurring within the different states sampled up to the micro- to millisecond range, such that ⟨*R*⟩ = ∑_*i*_*p*_*i*_*R*^*i*^ (*p*_*i*_ and *R*^*i*^ are the population and the relaxation of each state). The problem of reproducing experimental relaxation rates from IDPs using MD simulation is illustrated in Figure 6, where the 18 rates from Sendai virus NT are compared to those derived from several microseconds of fully solvated trajectories, using (in 2016) state-of-the-art, IDP-adapted force fields.^90,258^

Analysis of these trajectories indicates that the origin of the discrepancy derived from the over-representation of rare events, such as long-range contacts, whose frequency is poorly sampled, leading to statistical instability because the sampled correlation time does not fulfill the necessary criterion *τ*_*eff*_ ≪ *t*_max_,^259^ where *t*_max_ is the maximal sampled time of the angular correlation function. To address this problem, the following procedure was adopted: The entire trajectory, or multiple distinct trajectories nucleated from different conformations, are divided into subtrajectories of 100 ns, from which correlation functions *C*_*i*_(*τ*) (and rates *R*^*i*^) are calculated and combined in an ensemble average that explicitly mimics the actual heterogeneous conformational origin of the measured relaxation. The maximum length of each subtrajectory is dictated according to the experimental analysis described above for the studies of two IDPs, NT and MKK4. At *T* = 298 K, the slowest contribution to the rotational correlation function detected by experimental spin relaxation (see above) is approximately 5 ns, so that the dynamic reorientations occurring in each distinct substate can be reasonably sampled using a sampling window of 100 ns (*t*_max_= 50 ns). The ABSURD (average block selection using relaxation data) approach then estimates the relative weights or segments of *C*_*i*_(*τ*) with respect to a single experimental relaxation rate, compiling an ensemble of subtrajectories that interchange on time scales significantly slower than the correlation time limit (100 ns) and significantly faster than the chemical shift time scale (100s of μs).^125^ In this way, a representative ensemble of time-dependent trajectories is identified, thereby extending the concept of conformationally averaged ensemble-descriptions into the time dimension. Optimization against a unique relaxation rate at a single field identifies an ensemble of trajectories that systematically improves agreement with a broad set of rates, sensitive to motions occurring on a range of time scales (*R*_*1*_, *R*_*2*_, *σ*_*NH*_, *η*_*z*_ measured at multiple fields) (Figure 6), as well as local (^13^C chemical shift) and global (SAXS) conformational sampling properties.

The fact that the ensemble of trajectories improves reproduction of “passive” dynamic reporters highlights the importance of correctly sampling the free energy landscape of the IDP in solution, and illustrates the complex interdependence of motions occurring on time scales varying over many orders of magnitude. While it has previously been shown that simulating motions occurring in distinct substates improves reproduction of relaxation in folded proteins,^119,260^ it is challenging to make this observation for IDPs.^120^

### 5.2 Analytical Description of the Dynamics of IDPs Sampled by NMR Relaxation

**Figure 7:**
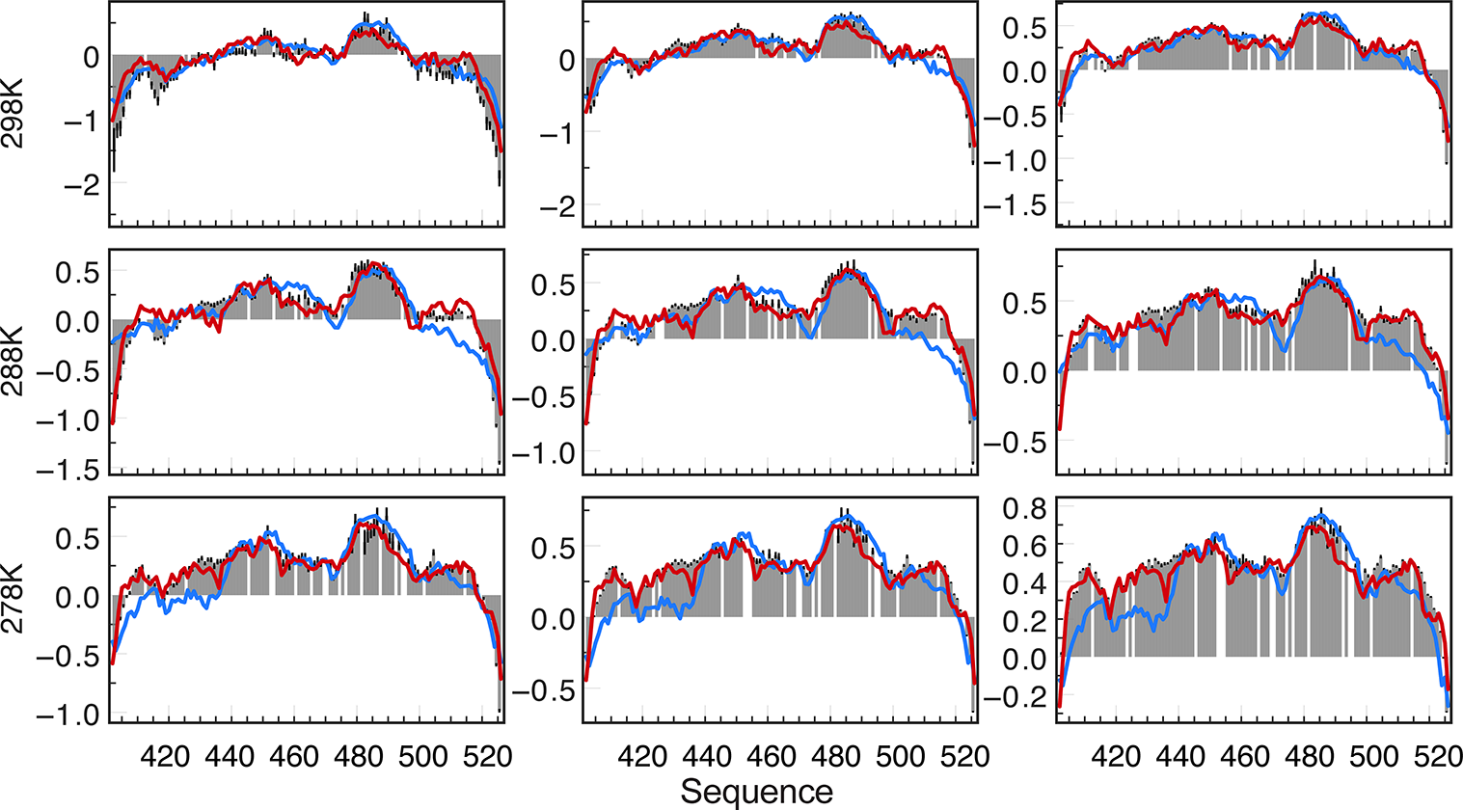
Temperature-dependent NMR relaxation identifies accurate and transferable molecular force fields for IDPs. Experimental ^15^N{^1^H} steady-state nOes (gray bars) measured on Sendai virus NT at different magnetic fields (left 600 MHz, middle 700 MHz, and right 850 MHz) and temperatures. ABSURD-selected ensembles of trajectories using Charmm36m combined with the TIP4*P*/2005 water model (red) reproduces experimental values better than when combined with TIP3P (blue), at all temperatures. (Reproduced with permission from Salvi et al. Sci. Adv. 2019^125^ Copyright 2019 AAAS).

The ability to simulate the ensemble averaged angular correlation functions is of course only half of the challenge. In principle this function describes all of the molecular mechanisms that are relaxation-active, but in practice it is not straightforward to extract motional modes from this complex function. To address this problem, the correlation function was recently analytically decomposed into three components using internal coordinates to describe librational and reorientational dihedral angle modes relative to the average peptide plane, and tumbling of each peptide relative to the laboratory frame.^257^ This deconvolution of the angular components allowed the identification of locally correlated and segmental motions along the chain. The advantage of such an approach was exemplified in a comparison of temperature dependent ^15^N relaxation measured on Sendai virus NT, and compared to relaxation calculated from average correlation functions derived using different force fields.^261^ This allowed the identification of the best force field over a range of temperatures (Figure 7) but also the exact dynamic mode that was responsible for the incorrect reproduction of experimental data (in this case the reorientation of water molecules and their correlation with intrasegmental backbone motions). In this way, the combination of ABSURD and the analytical description of the correlation functions can be seen as a forensic tool to improve molecular dynamics force fields with respect to experimental data.

## 6 How Do IDPs Function? Time-Resolved Atomic Resolution Descriptions of IDP Complexes

The detailed study of IDP-binding to receptors and cofactors has revealed that IDP-based affinities range from tight subnanomolar binding of highly specific chaperone complexes to multivalent interactions with individual dissociation constants in the millimolar range.^262−267^ NMR spectroscopy has the immense benefit of providing residue- or even atomic-resolution detail of the interaction trajectories of IDPs, even in the weak binding regime, and it is in this range of affinities that it most often provides unique functional insight.

Depending on the exchange regime between free and bound protein, NMR chemical shifts report on the population-weighted average of the free and bound forms of the protein (fast exchange, where the exchange occurs at a rate faster than the difference in chemical shifts ΔΔω in the two states) or slow exchange, that in principle allows for simultaneous detection of both environments.

The former regime has been elegantly exploited by Brüschweiler et al. to investigate the binding modes of different amino acids present in disordered proteins by measuring the impact of aqueous colloidal dispersions of anionic silica nanoparticles on the transverse relaxation rates of IDPs.^268,269^ Electrostatic and hydrophobic interactions are thought to dominate these weak interactions, and these are shown to differ largely between amino acid types. The authors show that these interactions can be parametrized and the binding profile of a given IDP can be accurately predicted using a simple mathematical model. This method also has the considerable advantage that transverse relaxation rates are impacted by motions occurring on time scales that are normally difficult to access by solution state NMR, also providing insight into the intrinsic dynamics of folded proteins.^270^

Beyond the fast exchange limit, intermediate exchange, occurring on time scales that are comparable to ΔΔω, leads to line-broadening of the observable peaks (Figure 1). This latter regime can be particularly informative because NMR exchange spectroscopy can be used to unravel the molecular mechanisms responsible for the observed broadening, even at very low population of bound state, simultaneously providing information both about the exchange kinetics and the free energy surface of the exchanging environments. Rotating frame relaxation (*R*_1ρ_),^134,135^ Carr–Purcell–Meiboom–Gill (CPMG) relaxation dispersion,^131,132,136^ chemical exchange saturation transfer (CEST),^133,271,272^ and zz-exchange^273,274^ provide information about exchange processes from the tens of microseconds to the subsecond range.

### 6.1 Describing the Interaction Trajectories of IDPs with Their Partner Proteins

**Figure 8:**
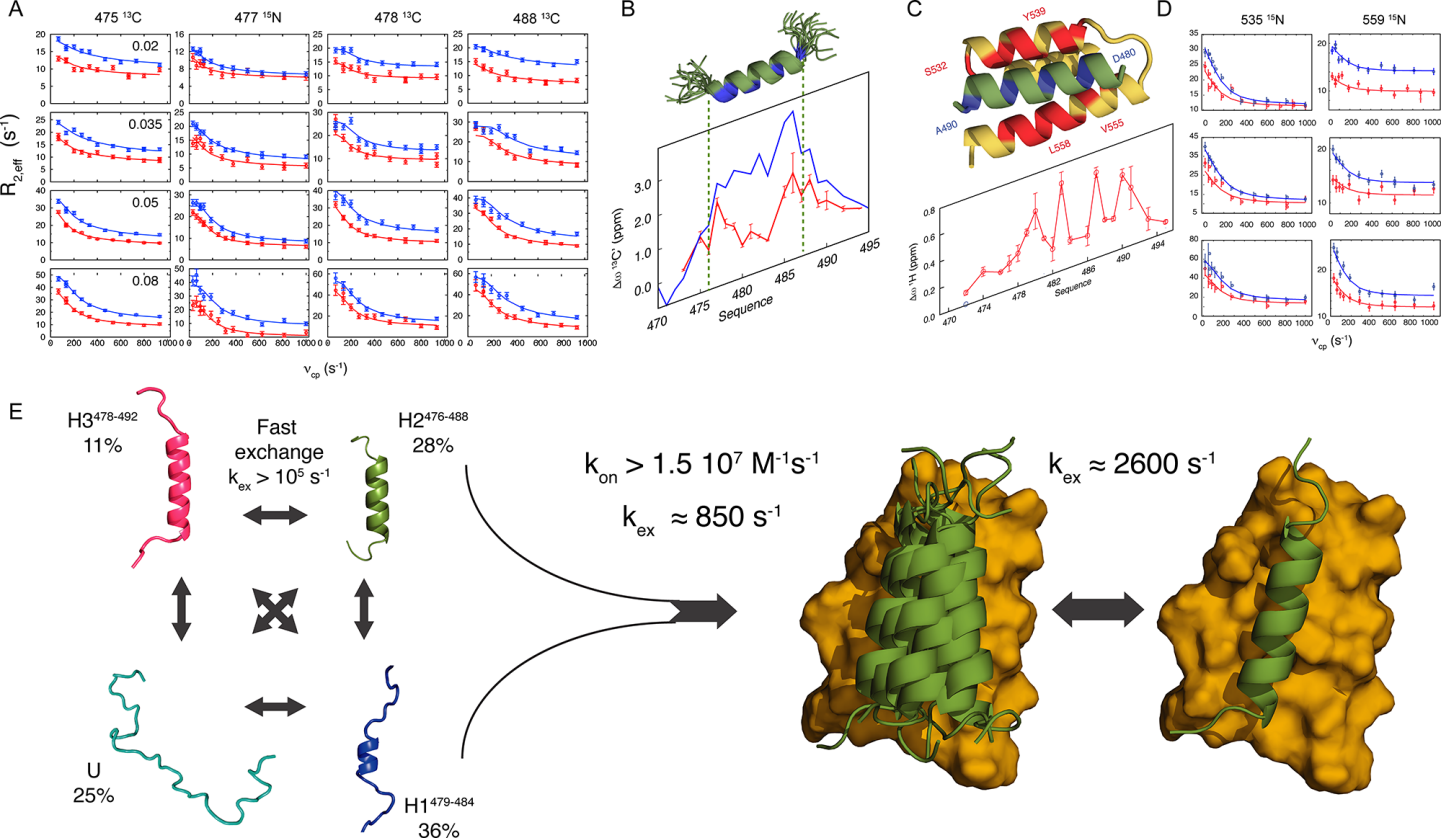
Multinuclear CPMG relaxation dispersion maps the molecular recognition trajectory of an intrinsically disordered protein as it binds its physiological partner. (A) ^1^H, ^13^C, and ^15^N CPMG were used to map the interaction trajectory of Sendai virus NT with the C-terminal domain of the phosphoprotein (PX). The combination of multinuclear CPMG, measured at multiple substoichiometric admixtures (2, 3.5, 5, and 8% of PX compared to NT) provides the necessary information to reconstruct a complex interaction trajectory involving both folding and binding. (B) The first step involves funnelling of one of the helical elements present in the equilibrium of rapidly exchanging substates, in an encounter complex on the surface of PX. (C) The second step involves binding of the stabilized helix into a groove between two helices on the surface of PX. (D) Relaxation dispersion measured on NT confirms that the second step coincides with events occurring on the surface of NT. (E) Representation of the most likely interaction trajectory derived from the ensemble of the experimental data. (Reproduced with permission from Schneider et al. JACS 2015^191^ Copyright 2015 American Chemical Society).

The power of CPMG relaxation dispersion to describe complex interaction trajectories of IDPs was demonstrated by Sugase et al.,^182^ who studied the interaction between the KIX domain of CREB binding protein and the phosphorylated form of kinase inducible activation domain (pKID). ^15^N CPMG measurements in the presence of substoichiometric admixtures of KIX provided evidence for weak binding between pKID and KIX, and allowed the authors to propose a model of the binding trajectory according to a three-site exchange model, describing binding via a partially folded encounter complex. This approach has been further exploited, using a combination of ^1^H, ^13^C, and ^15^N CPMG, to map the interaction trajectory of Sendai NT upon binding to the C-terminal domain of the phosphoprotein (PX).^191^ While ^1^H and ^15^N amide chemical shifts are commonly used as probes to map interaction interfaces, ^13^C backbone chemical shifts are more sensitive to secondary structure. ^1^H, ^13^C, and ^15^N CPMG, measured at substoichiometric admixtures of PX, was used to map the conformational transitions along the interaction trajectory of the partially formed helical motif (Figure 8). This motif had previously been characterized on the basis of RDCs and chemical shifts as a rapidly exchanging ensemble of distinct helical elements.^275^ The initial step of the interaction involves the stabilization of one of the helical elements present in the free-state equilibrium in an encounter complex on the surface of PX. This step is mainly characterized by ^13^Ć differences between the free state and the encounter complex. The second and final step, as reported mainly by ^1^H and ^15^N shifts, involves binding of the stabilized NT helix into a groove between two helices on the surface of PX. The combination of multinuclear CPMG, measurements on both partners and at multiple admixtures thus provides the necessary information to reconstruct a complex interaction trajectory involving both folding and binding. This study also highlights the importance of the intrinsic conformational dynamics of the binding partners that is already present in their free states. The conformational equilibrium of free NT comprises a pre-existing population of the state that is stabilized in the encounter complex, while the second binding step appears to be limited by breathing motions that open and close the binding pocket on PX in its free form.^108^ This example also demonstrates that simple models of intermolecular interaction such as “induced-fit” or “conformational selection” are not necessarily applicable to interactions involving highly dynamic proteins such as IDPs, where a broader terminology, for example, conformational funneling, would be necessary to describe such multistate interaction trajectories.^192^

The crowded environment of living cells can clearly influence interactions involving IDPs,^255,276,277^ impacting association and dissociation rates, via nonspecific interactions or modulation of the structural and dynamic behavior of the proteins described above. Although fluorescence^278^ and simulation has provided useful insight, for example, into the potential impact of attractive and repulsive interactions with the cellular milieu on coupled folding and binding,^279^ atomic or residue-specific experimental characterizations of IDP-mediated interactions *in vivo* remain relatively rare.^198,280−282^

To achieve a deeper understanding of the effects of crowding on the thermodynamics and kinetics of reactions involving IDPs and their partners, a more detailed, residue-specific picture is required, for example, using relaxation and exchange measurements in crowded environments and living cells. Kay and co-workers already performed ^15^N *R*_1ρ_ relaxation dispersion experiment in a highly concentrated phase-separated state (which can be regarded as a particular form of crowding) of the germ granule protein Ddx4, discovering a slowly exchanging excited state with increased intermolecular contacts.^283^

### 6.2 On the Importance of Multivalent, Weak Interactions in Biology

It is becoming increasingly clear that not all IDPs fold upon binding to their partners, even locally. The nuclear pore is filled with proteins (FG-nucleoporins) comprising extremely long IDRs, that are decorated with phenylalanine-glycine (FG) motifs, that control transition between the cytoplasm and the nucleoplasm. Larger proteins can only pass the filter when bound to nuclear transport receptors (NTRs). Despite the high selectivity of the filter, transport across the pore is extremely fast. The crucial interaction between NTRs and FG motifs was recently investigated using NMR, revealing weak chemical shift perturbations in the nucleoporin Nup153 in the presence of a series of NTRs.^68^ In this case, ^15^N R_1ρ_ and chemical shift titration confirmed that the interaction was in fast exchange, allowing an estimate of the intrinsic individual dissociation constant of a single site of around 8 mM. The presence of multiple motifs in a single protein clearly illustrated the effect of multivalency on the apparent affinity, which decreased with increasing multivalency. Finally, assignment of both free and bound forms of Nup153 demonstrated a complete absence of backbone conformational transition upon binding, with the disordered domain maintaining a high level of plasticity in the complex. On the basis of these results, a model was proposed of rapid passage, assured by the quasi continuum of NTR-binding sites present throughout the pore, and the fast on and off rates that are maintained by multivalent ultraweak binding throughout this continuum. Related results were also found for other nucleoporins,^284,285^ suggesting that the mechanism may be general.

**Figure 9:**
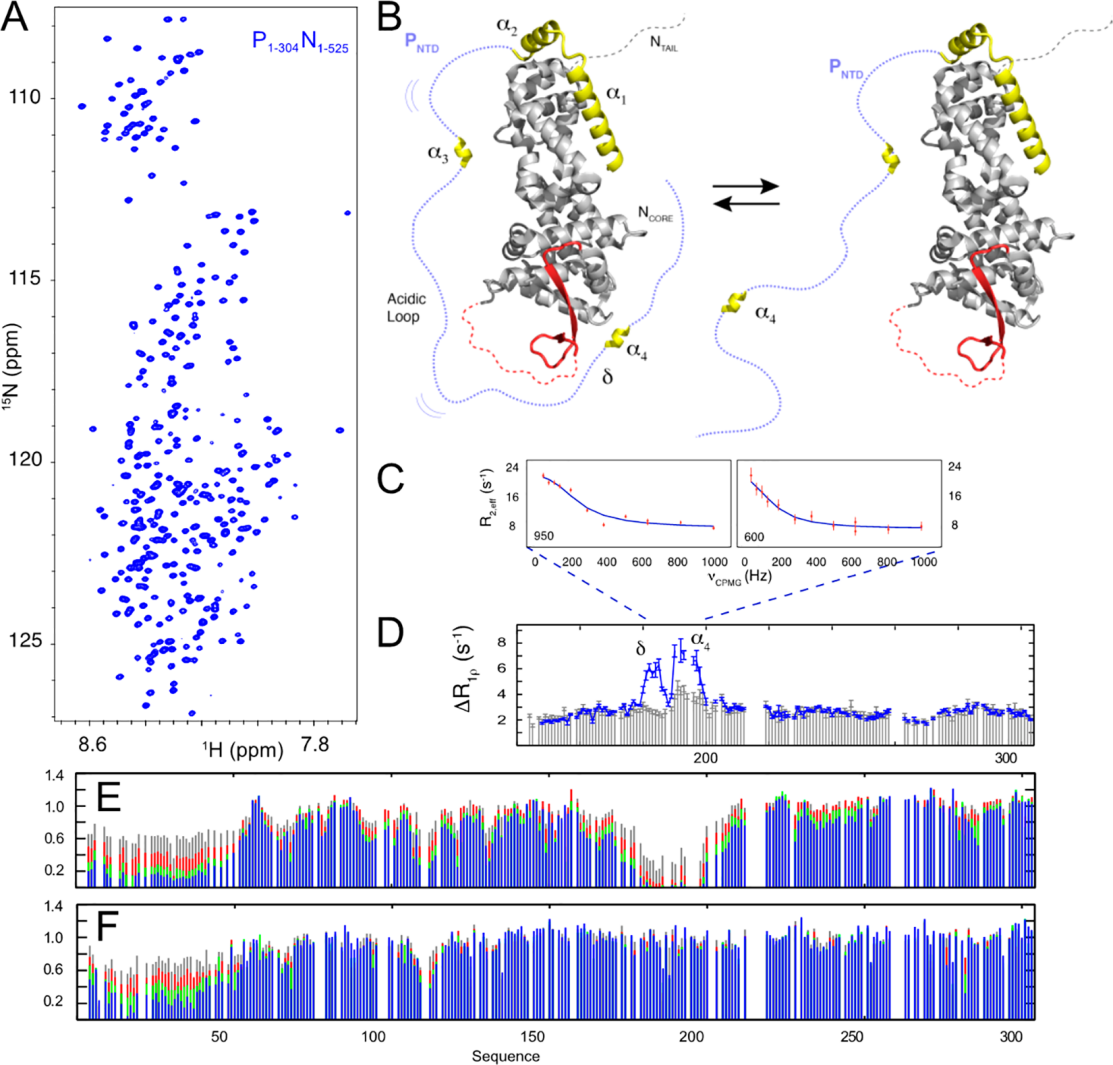
NMR detects essential, ultraweak interactions in the dynamic assembly of Measles virus nucleo/phosphoprotein complex. (A) ^15^N–^1^H HSQC spectrum of the complex formed between P_TAIL_ and the nucleoprotein. The complex comprises more than 450 intrinsically disordered amino acids. (B) Representation of the two interaction sites involved in the complex. The phosphoprotein of Measles virus (yellow) is known to bind the nucleoprotein (gray) in a tight complex at its N-terminal end. NMR reveals a second binding site (δα4) that is 150 amino acids away from the first binding site, in the middle of a long intrinsically disordered domain that binds a distal site of the nucleoprotein. NMR exchange (C) ^15^N CPMG and (D) rotating frame relaxation in the free and bound forms of the region 140–304 of P_TAIL_, reveals that the intrinsic affinity of this second site is 5 orders of magnitude lower than the known binding site. (E) Normalized peak intensities (I/I_0_) of P_1–304_ (50 μM) with P_1–50_N_1–525_ (gray, 25; red, 50; green, 100; and blue, 150 μM concentrations of P_1–304_. (F) Interaction profile of P_1–304_,HELL → AAAA mutation (concentrations as in E). Mutation of these four residues in the binding site knocks out the second interaction and replication. (Reproduced with permission from Milles et al. Sci. Adv. 2018^286^ Copyright 2018 AAAS).

Another example of the physiological importance of ultraweak binding is shown from the study of the chaperone complex between the partially disordered nucleoprotein (N) and the intrinsically disordered phosphoprotein of Measles virus (MeV).^286^ Paramyxoviral phosphoproteins (P) are essential cofactors of the replication complex: they are tetrameric and all comprise long IDRs that are hundreds of amino acids in length and whose function remains largely unknown.^287^ N has a folded domain that encapsidates the viral genome, protecting it from the host immune system, and a disordered C-terminal domain. ASTEROIDS analysis of the 304 amino acid IDR of P from MeV identifies short helical elements in the N-terminal domain, and an additional fourth helix 150 amino acids downstream of this (α_4_), adjacent to a highly acidic strand. The N-terminal helices bind tightly to N, maintaining it in its monomeric form prior to encapsidation of the RNA genome. The 90 kDa NP complex was investigated using NMR, including over 450 intrinsically disordered residues, identifying the known N-terminal chaperone binding site, but also a second, previously unknown binding site positioned at the fourth helical element, α_4_ (Figure 9). ^15^N CPMG using a molecular construct comprising only this site revealed that the interaction has an intrinsic affinity that is around 5 orders of magnitude weaker than the main interaction site, allowing P to transiently wrap around N, and to exchange between compact and extended forms. Remarkably, the conserved interaction motif is shown to be essential for viral replication. Although the exact role of the second binding site remains unknown, it is possible that conformational fluctuations of the acidic loop between the binding sites on P frustrate access to the surface of N, for example, by cellular RNA or inhibit self-assembly with other N monomers. More generally, the combination of two distant interactions involving the same IDR suggests the existence of long-range coupling between the two interaction sites linking opposite ends of N that is regulated by the highly disordered nature of P. This example again highlights the extreme sensitivity of NMR to detect ultraweak interactions, even in the presence of very strong affinity interactions between the same partners.

### 6.3 Atomic Resolution Descriptions of Highly Dynamic Molecular Assemblies from NMR

Disordered domains are thought to play a role in the replication of numerous single strand RNA viruses, with components of the replication machinery from both negative^287,288^ and positive sense^289−293^ RNA viruses exhibiting extensive disorder. A recent description of the nucleoprotein of SARS-CoV-2, involved in protection of the viral genome and regulation of gene transcription, revealed that the flexible central region undergoes a disorder to order transition, folding around the N-terminal domain of its viral partner nsp3 and inducing a collapse of the remainder of the protein that impacts its ability to bind RNA.^294^

Influenza A represents another example where extreme disorder appears to play an essential role in viral function. To efficiently replicate in human cells, avian influenza polymerase undergoes host adaptation, with adaptive mutants (in particular E627 K) localized on two C-terminal (627 and NLS) domains of the PB2 polymerase subunit. This region of the protein shows remarkable behavior in solution, populating an equilibrium between open and closed conformations that can be characterized using ^15^N CEST experiments, revealing open form chemical shifts that are essentially identical to the isolated domains in free solution and determine the exchange rate to be around 20 s^–1^.^295^ The closed form is stabilized by an interdomain salt bridge^296^ while in the open form the linker connecting the two domains becomes highly dynamic and the two domains evolve freely. The host transcription factor ANP32a was identified as an essential cofactor for the adaptation of the viral polymerase,^297^ suggesting a direct interaction between the two proteins. ANP32a has a highly acidic, intrinsically disordered domain whose length varies between species, with the avian form containing a 33 amino acid insert, comprising a unique hydrophobic hexapeptide and a repeat of the first 27 acidic amino acids. Somehow the absence of this insert in mammals is compensated by a single E627 K mutation of the avian polymerase, allowing cross-species infection. It was therefore important to investigate the complexes between these two highly flexible proteins.

Here again, the IDR mediates the interaction, with a polyvalent interaction between the acidic tail of ANP32a and the positively charged surface of the 627 domain.^298^ The intrinsic *K*_D_ measured from the side of ANP32a is more than 1 order of magnitude lower than the *K*_D_ measured from the side of 627 due to the multiple interaction sites on ANP32a dispersed along the IDR visiting the same sites on 627-NLS. To characterize the dynamic ensembles, a series of eight cysteine mutants of the avian and human adapted forms of 627-NLS were made, and PREs measured on ANP32a. In the fast exchange regime, these data provide a sensitive map of the population-weighted proximity of the two proteins over the dynamic assembly and were used to develop an ensemble description of the human and avian complexes using the ASTEROIDS ensemble approach.

**Figure 10:**
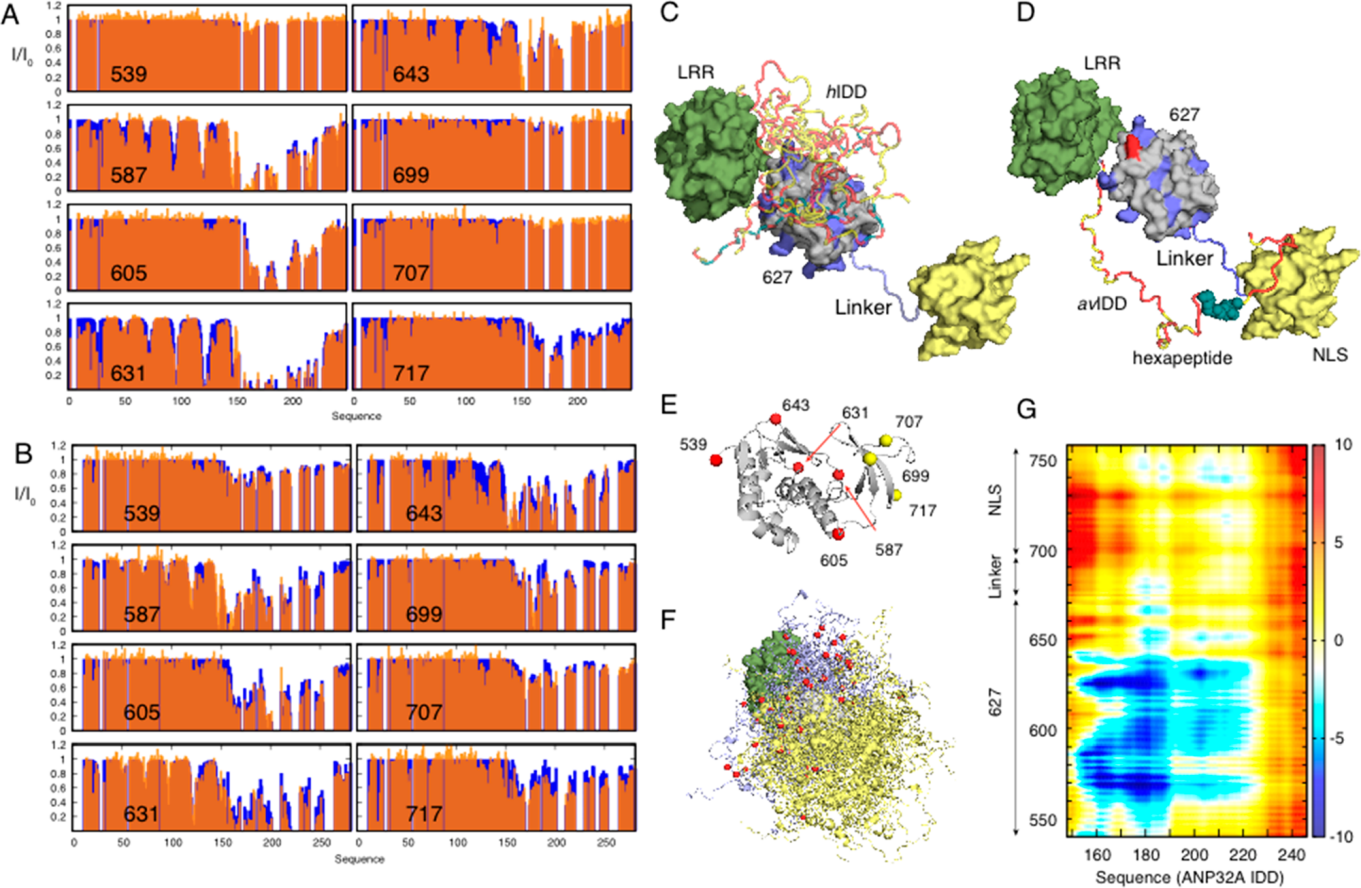
Influenza polymerase forms a highly dynamic assembly with the intrinsically disordered host transcription factor ANP32a in a species specific-way. (A) PREs measured on *h*ANP32A (orange, experimental; and blue, representative ensembles selected using ASTEROIDS) in the presence of paramagnetically labeled human adapted 627-NLS. (B) Same information for *av*ANP32A in the presence of paramagnetically labeled avian adapted 627-NLS. (C, D) Representation of the dynamic complexes determined from the data shown in A and B, respectively. Multivalent interactions between ANP32a (yellow/red) and the 627 domain (gray) are localized to the basic patch on the surface of 627. In the case of *av*ANP32A and avian adapted 627-NLS(E), ANP32A disordered domain is in general closer to the NLS domain (yellow) mediated by the hydrophobic hexapeptide (green). (E) Position of the cysteine residues used to label 627-NLS. (F) Representation of the ensemble of conformers of the *h*ANP32A:627-NLS complex. (G) Average distance difference matrix (in Å) between ANP32A (*x*-axis) and the 627-NLS domains (*y*-axis) over the two ensembles. (Reproduced with permission from Camacho-Zarco et al. Nat. Commun. 2020^298^).

This comparison identifies clear distinctions between the binding modes exploited in the two complexes (Figure 10), as shown quantitatively in the average distance map, where closer or more populated contacts are observed between the positively charged 627 domain and the acidic IDR for the human complex than for the avian complex where the electrostatic distribution on the surface of 627 is disrupted by the E627 K mutation. This study allows us to speculate further on the role of the interaction in the function of the replication complex and more generally demonstrates the ability of NMR to characterize intermolecular complexes exhibiting extreme levels of flexibility and multivalency.

It is perhaps not surprising that electrostatic interactions in low complexity IDPs can be responsible for highly multivalent interactions. This was clearly demonstrated by a combination of smFRET and NMR spectroscopy, together with coarse grained MD simulation, to investigate the complex between two IDPs, the strongly basic histone H1 and the highly negatively charged prothymosin-α.^299^ Fluorescence spectroscopy reveals affinities in the picomolar range, while NMR and smFRET reveal that the proteins remain dynamic within the complex, implying a high level of dynamic polyvalency and possible formation of transient ternary complexes.^300^ The presence of dynamics in the bound state of IDRs was also characterized in two recent studies of the disordered domain of kinases MKK7,^301^ MKK4^302,303^ in complex with JNK1 and p38α. CEST, CPMG, and spin relaxation were measured as a function of stoichiometric ratio, suggesting that the bound state of MKK7, and the kinase specificity regions flanking the main interaction site of MKK4, both exhibited additional dynamics in the bound state, in the former case on the micro to millisecond time scale and the latter on relaxation-active ps-ns time scales. Similar data were used to investigate the interaction between Artemis and the DNA binding domain of ligase IV, in this case identifying a single step binding interaction.^304^

## 7 Perspectives

Over the course of this review, we have demonstrated the unique insight that NMR offers concerning the structure, dynamics and interactions of IDPs at atomic resolution not only in reduced systems comprising isolated proteins but also in the context of more complex molecular environments that are relevant to physiological function. In particular, we have drawn attention to the importance of describing the ensemble and time-averaging processes that govern interpretation of NMR parameters, and the remarkable insight that this can provide concerning the functional modes exploited by such highly dynamic systems. The power of NMR results in part from analytical understanding of the ensemble and time-averaging processes occurring on time scales covering orders of magnitude from pico- to milliseconds that remains one of its unique advantages for studying flexible molecules. In addition to providing unique new insight into the relationship between protein flexibility and function, the combination of atomic resolution characterization of essential dynamic processes from NMR with complementary structural and dynamic probes that can be measured on similar sample preparations ensures an exciting future for NMR as an integral tool for the investigation of increasingly complex biological systems.

## Abbreviations

NMR: nuclear magnetic resonance
CEST: chemical exchange saturation transfer
CPMG: Carr-Purcell-Meiboon and Gill
IDP: intrinsically disordered protein
IDR: intrinsically disordered region
MKK4: mitogen-activated protein kinase kinase 4
MKK7: mitogen-activated protein kinase kinase 7
smFRET: single molecule Förster resonance energy transfer
ABSURD: average block selection using relaxation data
ASTEROIDS: a selection tool for ensemble representation of intrinsically disordered systems
RNAP: ribose-nucleic acid polymerase
ANP32a: acidic leucine-rich nuclear phosphoprotein 32 (family member A)
NT: C-terminal domain of paramyxoviral nucleoprotein
PX: C-terminal domain of paramyxoviral phosphoprotein
KIX: kinase-inducible domain (KID) interacting domain
KID: kinase inducible activation domain
NLS: nuclear localization signal
CSA: chemical shift anisotropy
PRE: paramagnetic relaxation enhancement
RDC: residual dipolar couplings
SAXS: small angle X-ray scattering
MD: molecular dynamics
UV: ultraviolet
IR: infrared

## References

[ref1] Uversky V. N. Natively Unfolded Proteins: A Point Where Biology Waits for Physics. Protein Sci. 2002, 11, 739–756. 10.1110/ps.4210102. 11910019 PMC2373528

[ref2] Tompa P. Intrinsically Unstructured Proteins. Trends Biochem. Sci. 2002, 27, 527–533. 10.1016/S0968-0004(02)02169-2. 12368089

[ref3] Dyson H. J.; Wright P. E. Intrinsically Unstructured Proteins and Their Functions. Nat. Rev. Mol. Cell Biol. 2005, 6, 197–208. 10.1038/nrm1589. 15738986

[ref4] Uversky V. N.; Dunker A. K. Understanding Protein Non-Folding. Biochim. Biophys. Acta 2010, 1804, 1231–1264. 10.1016/j.bbapap.2010.01.017. 20117254 PMC2882790

[ref5] Tompa P.; Davey N. E.; Gibson T. J.; Babu M. M. A Million Peptide Motifs for the Molecular Biologist. Mol. Cell 2014, 55, 161–169. 10.1016/j.molcel.2014.05.032. 25038412

[ref6] Csermely P.; Palotai R.; Nussinov R. Induced Fit, Conformational Selection and Independent Dynamic Segments: An Extended View of Binding Events. Trends Biochem. Sci. 2010, 35, 539–546. 10.1016/j.tibs.2010.04.009. 20541943 PMC3018770

[ref7] Shin Y.; Brangwynne C. P. Liquid Phase Condensation in Cell Physiology and Disease. Science 2017, 357, eaaf4382 10.1126/science.aaf4382. 28935776

[ref8] Lim M. H.; Jackson T. A.; Anfinrud P. A. Ultrafast Rotation and Trapping of Carbon Monoxide Dissociated from Myoglobin. Nat. Struct. Biol. 1997, 4, 209–214. 10.1038/nsb0397-209. 9164462

[ref9] Thielges M. C.; Fayer M. D. Protein Dynamics Studied with Ultrafast Two-Dimensional Infrared Vibrational Echo Spectroscopy. Acc. Chem. Res. 2012, 45, 1866–1874. 10.1021/ar200275k. 22433178 PMC3389584

[ref10] Ebbinghaus S.; Kim S. J.; Heyden M.; Yu X.; Heugen U.; Gruebele M.; Leitner D. M.; Havenith M. An Extended Dynamical Hydration Shell around Proteins. Proc. Natl. Acad. Sci. U. S. A. 2007, 104, 20749–20752. 10.1073/pnas.0709207104. 18093918 PMC2410073

[ref11] Doster W.; Cusack S.; Petry W. Dynamical Transition of Myoglobin Revealed by Inelastic Neutron Scattering. Nature 1989, 337, 754–756. 10.1038/337754a0. 2918910

[ref12] Cametti C.; Marchetti S.; Gambi C. M. C.; Onori G. Dielectric Relaxation Spectroscopy of Lysozyme Aqueous Solutions: Analysis of the δ-Dispersion and the Contribution of the Hydration Water. J. Phys. Chem. B 2011, 115, 7144–7153. 10.1021/jp2019389. 21557554

[ref13] Frauenfelder H.; Sligar S. G.; Wolynes P. G. The Energy Landscapes and Motions of Proteins. Science 1991, 254, 1598–1603. 10.1126/science.1749933. 1749933

[ref14] Buhrke D.; Hildebrandt P. Probing Structure and Reaction Dynamics of Proteins Using Time-Resolved Resonance Raman Spectroscopy. Chem. Rev. 2020, 120, 3577–3630. 10.1021/acs.chemrev.9b00429. 31814387

[ref15] Schotte F.; Cho H. S.; Kaila V. R. I.; Kamikubo H.; Dashdorj N.; Henry E. R.; Graber T. J.; Henning R.; Wulff M.; Hummer G.; et al. Watching a Signaling Protein Function in Real Time via 100-Ps Time-Resolved Laue Crystallography. Proc. Natl. Acad. Sci. U.S.A. 2012, 109, 19256–19261. 10.1073/pnas.1210938109. 23132943 PMC3511082

[ref16] Tenboer J.; Basu S.; Zatsepin N.; Pande K.; Milathianaki D.; Frank M.; Hunter M.; Boutet S.; Williams G. J.; Koglin J. E.; et al. Time-Resolved Serial Crystallography Captures High-Resolution Intermediates of Photoactive Yellow Protein. Science 2014, 346, 1242–1246. 10.1126/science.1259357. 25477465 PMC4361027

[ref17] Dyson H. J.; Wright P. E. Unfolded Proteins and Protein Folding Studied by NMR. Chem. Rev. 2004, 104, 3607–3622. 10.1021/cr030403s. 15303830

[ref18] Fink A. L. Natively Unfolded Proteins. Curr. Opin. Struct. Biol. 2005, 15, 35–41. 10.1016/j.sbi.2005.01.002. 15718131

[ref19] Mittag T.; Forman-Kay J. D. Atomic-Level Characterization of Disordered Protein Ensembles. Curr. Opin. Struct. Biol. 2007, 17, 3–14. 10.1016/j.sbi.2007.01.009. 17250999

[ref20] Dunker A. K.; Silman I.; Uversky V. N.; Sussman J. L. Function and Structure of Inherently Disordered Proteins. Curr. Opin. Struct. Biol. 2008, 18, 756–764. 10.1016/j.sbi.2008.10.002. 18952168

[ref21] Tompa P.; Fuxreiter M. Fuzzy Complexes: Polymorphism and Structural Disorder in Protein-Protein Interactions. Trends Biochem. Sci. 2008, 33, 2–8. 10.1016/j.tibs.2007.10.003. 18054235

[ref22] Eliezer D. Biophysical Characterization of Intrinsically Disordered Proteins. Curr. Opin. Struct. Biol. 2009, 19, 23–30. 10.1016/j.sbi.2008.12.004. 19162471 PMC2728036

[ref23] Wright P. E.; Dyson H. J. Linking Folding and Binding. Curr. Opin. Struct. Biol. 2009, 19, 31–38. 10.1016/j.sbi.2008.12.003. 19157855 PMC2675572

[ref24] Fisher C. K.; Stultz C. M. Constructing Ensembles for Intrinsically Disordered Proteins. Curr. Opin. Struct. Biol. 2011, 21, 426–431. 10.1016/j.sbi.2011.04.001. 21530234 PMC3112268

[ref25] Van Roey K.; Gibson T. J.; Davey N. E. Motif Switches: Decision-Making in Cell Regulation. Curr. Opin. Struct. Biol. 2012, 22, 378–385. 10.1016/j.sbi.2012.03.004. 22480932

[ref26] Forman-Kay J. D.; Mittag T. From Sequence and Forces to Structure, Function, and Evolution of Intrinsically Disordered Proteins. Structure 2013, 21, 1492–1499. 10.1016/j.str.2013.08.001. 24010708 PMC4704097

[ref27] Kosol S.; Contreras-Martos S.; Cedeño C.; Tompa P. Structural Characterization of Intrinsically Disordered Proteins by NMR Spectroscopy. Molecules 2013, 18, 10802–10828. 10.3390/molecules180910802. 24008243 PMC6269831

[ref28] Bernado P.; Mylonas E.; Petoukhov M. V.; Blackledge M.; Svergun D. I. Structural Characterization of Flexible Proteins Using Small-Angle X-Ray Scattering. J. Am. Chem. Soc. 2007, 129, 5656–5664. 10.1021/ja069124n. 17411046

[ref29] Aznauryan M.; Delgado L.; Soranno A.; Nettels D.; Huang J.-R.; Labhardt A. M.; Grzesiek S.; Schuler B. Comprehensive Structural and Dynamical View of an Unfolded Protein from the Combination of Single-Molecule FRET, NMR, and SAXS. Proc. Natl. Acad. Sci. U.S.A. 2016, 113, E5389–5398. 10.1073/pnas.1607193113. 27566405 PMC5027429

[ref30] Fuertes G.; Banterle N.; Ruff K. M.; Chowdhury A.; Mercadante D.; Koehler C.; Kachala M.; Estrada Girona G.; Milles S.; Mishra A.; et al. Decoupling of Size and Shape Fluctuations in Heteropolymeric Sequences Reconciles Discrepancies in SAXS vs. FRET Measurements. Proc. Natl. Acad. Sci. U.S.A. 2017, 114, E6342–E6351. 10.1073/pnas.1704692114. 28716919 PMC5547626

[ref31] Riback J. A.; Bowman M. A.; Zmyslowski A. M.; Knoverek C. R.; Jumper J. M.; Hinshaw J. R.; Kaye E. B.; Freed K. F.; Clark P. L.; Sosnick T. R. Innovative Scattering Analysis Shows That Hydrophobic Disordered Proteins Are Expanded in Water. Science 2017, 358, 238–241. 10.1126/science.aan5774. 29026044 PMC5959285

[ref32] Gomes G.-N. W.; Krzeminski M.; Namini A.; Martin E. W.; Mittag T.; Head-Gordon T.; Forman-Kay J. D.; Gradinaru C. C. Conformational Ensembles of an Intrinsically Disordered Protein Consistent with NMR, SAXS, and Single-Molecule FRET. J. Am. Chem. Soc. 2020, 142, 15697–15710. 10.1021/jacs.0c02088. 32840111 PMC9987321

[ref33] Marsh J. A.; Forman-Kay J. D. Structure and Disorder in an Unfolded State under Nondenaturing Conditions from Ensemble Models Consistent with a Large Number of Experimental Restraints. J. Mol. Biol. 2009, 391, 359–374. 10.1016/j.jmb.2009.06.001. 19501099

[ref34] Salmon L.; Nodet G.; Ozenne V.; Yin G.; Jensen M. R.; Zweckstetter M.; Blackledge M. NMR Characterization of Long-Range Order in Intrinsically Disordered Proteins. J. Am. Chem. Soc. 2010, 132, 8407–8418. 10.1021/ja101645g. 20499903

[ref35] Fisher C. K.; Huang A.; Stultz C. M. Modeling Intrinsically Disordered Proteins with Bayesian Statistics. J. Am. Chem. Soc. 2010, 132, 14919–14927. 10.1021/ja105832g. 20925316 PMC2956375

[ref36] Jensen M. R.; Blackledge M. Testing the Validity of Ensemble Descriptions of Intrinsically Disordered Proteins. Proc. Natl. Acad. Sci. U.S.A. 2014, 111, E1557 10.1073/pnas.1323876111. 24639541 PMC4000822

[ref37] Jensen M. R.; Zweckstetter M.; Huang J.; Blackledge M. Exploring Free-Energy Landscapes of Intrinsically Disordered Proteins at Atomic Resolution Using NMR Spectroscopy. Chem. Rev. 2014, 114, 6632–6660. 10.1021/cr400688u. 24725176

[ref38] De Simone A.; Richter B.; Salvatella X.; Vendruscolo M. Toward an Accurate Determination of Free Energy Landscapes in Solution States of Proteins. J. Am. Chem. Soc. 2009, 131, 3810–3811. 10.1021/ja8087295. 19292482

[ref39] Roux B.; Weare J. On the Statistical Equivalence of Restrained-Ensemble Simulations with the Maximum Entropy Method. J. Chem. Phys. 2013, 138 (8), 084107 10.1063/1.4792208. 23464140 PMC3598863

[ref40] Hummer G.; Köfinger J. Bayesian Ensemble Refinement by Replica Simulations and Reweighting. J. Chem. Phys. 2015, 143, 243150 10.1063/1.4937786. 26723635

[ref41] Bonomi M.; Heller G. T.; Camilloni C.; Vendruscolo M. Principles of Protein Structural Ensemble Determination. Curr. Opin. Struct. Biol. 2017, 42, 106–116. 10.1016/j.sbi.2016.12.004. 28063280

[ref42] Sgourakis N. G.; Yan Y.; McCallum S. A.; Wang C.; Garcia A. E. The Alzheimer’s Peptides A Beta 40 and 42 Adopt Distinct Conformations in Water: A Combined MD/NMR Study. J. Mol. Biol. 2007, 368, 1448–1457. 10.1016/j.jmb.2007.02.093. 17397862 PMC1978067

[ref43] Wu K.-P.; Weinstock D. S.; Narayanan C.; Levy R. M.; Baum J. Structural Reorganization of α-Synuclein at Low PH Observed by NMR and REMD Simulations. J. Mol. Biol. 2009, 391, 784–796. 10.1016/j.jmb.2009.06.063. 19576220 PMC2766395

[ref44] Terakawa T.; Takada S. Multiscale Ensemble Modeling of Intrinsically Disordered Proteins: P53 N-Terminal Domain. Biophys. J. 2011, 101, 1450–1458. 10.1016/j.bpj.2011.08.003. 21943426 PMC3177054

[ref45] Knott M.; Best R. B. A Preformed Binding Interface in the Unbound Ensemble of an Intrinsically Disordered Protein: Evidence from Molecular Simulations. PLoS Comput. Biol. 2012, 8, e1002605 10.1371/journal.pcbi.1002605. 22829760 PMC3400577

[ref46] Zhang W.; Ganguly D.; Chen J. Residual Structures, Conformational Fluctuations, and Electrostatic Interactions in the Synergistic Folding of Two Intrinsically Disordered Proteins. PLoS Comput. Biol. 2012, 8, e1002353 10.1371/journal.pcbi.1002353. 22253588 PMC3257294

[ref47] Narayanan C.; Weinstock D. S.; Wu K.-P.; Baum J.; Levy R. M. Investigation of the Polymeric Properties of Alpha-Synuclein and Comparison with NMR Experiments: A Replica Exchange Molecular Dynamics Study. J. Chem. Theory Comput. 2012, 8, 3929–3942. 10.1021/ct300241t. 23162382 PMC3496295

[ref48] Wang Y.; Chu X.; Longhi S.; Roche P.; Han W.; Wang E.; Wang J. Multiscaled Exploration of Coupled Folding and Binding of an Intrinsically Disordered Molecular Recognition Element in Measles Virus Nucleoprotein. Proc. Natl. Acad. Sci. U.S.A. 2013, 110, e3743 10.1073/pnas.1308381110. 24043820 PMC3791790

[ref49] Mittal J.; Yoo T. H.; Georgiou G.; Truskett T. M. Structural Ensemble of an Intrinsically Disordered Polypeptide. J. Phys. Chem. B 2013, 117, 118–124. 10.1021/jp308984e. 23205890

[ref50] Bonomi M.; Camilloni C.; Cavalli A.; Vendruscolo M. Metainference: A Bayesian Inference Method for Heterogeneous Systems. Sci. Adv. 2016, 2, e1501177 10.1126/sciadv.1501177. 26844300 PMC4737209

[ref51] Lincoff J.; Haghighatlari M.; Krzeminski M.; Teixeira J. M. C.; Gomes G.-N. W.; Gradinaru C. C.; Forman-Kay J. D.; Head-Gordon T. Extended Experimental Inferential Structure Determination Method in Determining the Structural Ensembles of Disordered Protein States. Commun. Chem. 2020, 3, 74 10.1038/s42004-020-0323-0. 32775701 PMC7409953

[ref52] Ozenne V.; Schneider R.; Yao M.; Huang J.-R.; Salmon L.; Zweckstetter M.; Jensen M. R.; Blackledge M. Mapping the Potential Energy Landscape of Intrinsically Disordered Proteins at Amino Acid Resolution. J. Am. Chem. Soc. 2012, 134, 15138–15148. 10.1021/ja306905s. 22901047

[ref53] Das R. K.; Ruff K. M.; Pappu R. V. Relating Sequence Encoded Information to Form and Function of Intrinsically Disordered Proteins. Curr. Opin. Struct. Biol. 2015, 32, 102–112. 10.1016/j.sbi.2015.03.008. 25863585 PMC4512920

[ref54] Huang C.-Y.; Getahun Z.; Zhu Y.; Klemke J. W.; DeGrado W. F.; Gai F. Helix Formation via Conformation Diffusion Search. PROC. NATL. ACAD. SCI. U.S.A. 2002, 99, 2788–2793. 10.1073/pnas.052700099. 11867741 PMC122426

[ref55] Hamm P.; Helbing J.; Bredenbeck J. Two-Dimensional Infrared Spectroscopy of Photoswitchable Peptides. In Annual Review of Physical Chemistry; Annual Reviews: Palo Alto, CA, 2008; Vol. 59, pp 291–317.

[ref56] Balakrishnan G.; Weeks C. L.; Ibrahim M.; Soldatova A. V.; Spiro T. G. Protein Dynamics from Time Resolved UV Raman Spectroscopy. Curr. Opin. Struct. Biol. 2008, 18, 623–629. 10.1016/j.sbi.2008.06.001. 18606227 PMC2583231

[ref57] Gallat F.-X.; Laganowsky A.; Wood K.; Gabel F.; van Eijck L.; Wuttke J.; Moulin M.; Haertlein M.; Eisenberg D.; Colletier J.-P.; Zaccai G.; Weik M. Dynamical Coupling of Intrinsically Disordered Proteins and Their Hydration Water: Comparison with Folded Soluble and Membrane Proteins. Biophys. J. 2012, 103, 129–136. 10.1016/j.bpj.2012.05.027. 22828339 PMC3388209

[ref58] Perticaroli S.; Nickels J. D.; Ehlers G.; Mamontov E.; Sokolov A. P. Dynamics and Rigidity in an Intrinsically Disordered Protein, Beta-Casein. J. Phys. Chem. B 2014, 118, 7317–7326. 10.1021/jp503788r. 24918971

[ref59] Schiro G.; Fichou Y.; Gallat F.-X.; Wood K.; Gabel F.; Moulin M.; Haertlein M.; Heyden M.; Colletier J.-P.; Orecchini A.; et al. Translational Diffusion of Hydration Water Correlates with Functional Motions in Folded and Intrinsically Disordered Proteins. Nat. Commun. 2015, 6, 6490 10.1038/ncomms7490. 25774711 PMC4382692

[ref60] Kuzmenkina E. V.; Heyes C. D.; Nienhaus G. U. Single-Molecule Forster Resonance Energy Transfer Study of Protein Dynamics under Denaturing Conditions. Proc. Natl. Acad. Sci. U. S. A. 2005, 102, 15471–15476. 10.1073/pnas.0507728102. 16221762 PMC1266141

[ref61] Doose S.; Neuweiler H.; Sauer M. Fluorescence Quenching by Photoinduced Electron Transfer: A Reporter for Conformational Dynamics of Macromolecules. ChemPhysChem 2009, 10, 1389–1398. 10.1002/cphc.200900238. 19475638

[ref62] Ferreon A. C. M.; Gambin Y.; Lemke E. A.; Deniz A. A. Interplay of Alpha-Synuclein Binding and Conformational Switching Probed by Single-Molecule Fluorescence. Proc. Natl. Acad. Sci. U.S.A. 2009, 106, 5645–5650. 10.1073/pnas.0809232106. 19293380 PMC2667048

[ref63] Nettels D.; Mueller-Spaeth S.; Kuester F.; Hofmann H.; Haenni D.; Rueegger S.; Reymond L.; Hoffmann A.; Kubelka J.; Heinz B.; et al. Single-Molecule Spectroscopy of the Temperature-Induced Collapse of Unfolded Proteins. Proc. Natl. Acad. Sci. U. S. A. 2009, 106, 20740–20745. 10.1073/pnas.0900622106. 19933333 PMC2791578

[ref64] Müller-Späth S.; Soranno A.; Hirschfeld V.; Hofmann H.; Rüegger S.; Reymond L.; Nettels D.; Schuler B. From the Cover: Charge Interactions Can Dominate the Dimensions of Intrinsically Disordered Proteins. Proc. Natl. Acad. Sci. U.S.A. 2010, 107, 14609–14614. 10.1073/pnas.1001743107. 20639465 PMC2930438

[ref65] Milles S.; Lemke E. A. Single Molecule Study of the Intrinsically Disordered FG-Repeat Nucleoporin 153. Biophys. J. 2011, 101, 1710–1719. 10.1016/j.bpj.2011.08.025. 21961597 PMC3183753

[ref66] Soranno A.; Buchli B.; Nettels D.; Cheng R. R.; Müller-Späth S.; Pfeil S. H.; Hoffmann A.; Lipman E. A.; Makarov D. E.; Schuler B. Quantifying Internal Friction in Unfolded and Intrinsically Disordered Proteins with Single-Molecule Spectroscopy. Proc. Natl. Acad. Sci. U. S. A. 2012, 109, 17800–17806. 10.1073/pnas.1117368109. 22492978 PMC3497802

[ref67] Schuler B.; Hofmann H. Single-Molecule Spectroscopy of Protein Folding Dynamics–Expanding Scope and Timescales. Curr. Opin. Struct. Biol. 2013, 23, 36–47. 10.1016/j.sbi.2012.10.008. 23312353

[ref68] Milles S.; Mercadante D.; Aramburu I. V.; Jensen M. R.; Banterle N.; Koehler C.; Tyagi S.; Clarke J.; Shammas S. L.; Blackledge M.; et al. Plasticity of an Ultrafast Interaction between Nucleoporins and Nuclear Transport Receptors. Cell 2015, 163, 734–745. 10.1016/j.cell.2015.09.047. 26456112 PMC4622936

[ref69] Otosu T.; Ishii K.; Tahara T. Microsecond Protein Dynamics Observed at the Single-Molecule Level. Nat. Commun. 2015, 6, 7685 10.1038/ncomms8685. 26151767 PMC4506535

[ref70] Columbus L.; Hubbell W. L. A New Spin on Protein Dynamics. Trends Biochem. Sci. 2002, 27, 288–295. 10.1016/S0968-0004(02)02095-9. 12069788

[ref71] Kavalenka A.; Urbancic I.; Belle V.; Rouger S.; Costanzo S.; Kure S.; Fournel A.; Longhi S.; Guigliarelli B.; Strancar J. Conformational Analysis of the Partially Disordered Measles Virus N-TAIL-XD Complex by SDSL EPR Spectroscopy. Biophys. J. 2010, 98, 1055–1064. 10.1016/j.bpj.2009.11.036. 20303863 PMC2849088

[ref72] Chui A. J.; Lopez C. J.; Brooks E. K.; Chua K. C.; Doupey T. G.; Foltz G. N.; Kamel J. G.; Larrosa E.; Sadiki A.; Bridges M. D. Multiple Structural States Exist Throughout the Helical Nucleation Sequence of the Intrinsically Disordered Protein Stathmin, As Reported by Electron Paramagnetic Resonance Spectroscopy. Biochemistry 2015, 54, 1717–1728. 10.1021/bi500894q. 25715079

[ref73] Gillespie J. R.; Shortle D. Characterization of Long-Range Structure in the Denatured State of Staphylococcal Nuclease. I. Paramagnetic Relaxation Enhancement by Nitroxide Spin Labels. J. Mol. Biol. 1997, 268, 158–169. 10.1006/jmbi.1997.0954. 9149149

[ref74] Eliezer D.; Yao J.; Dyson H. J.; Wright P. E. Structural and Dynamic Characterization of Partially Folded States of Apomyoglobin and Implications for Protein Folding. Nat. Struct. Biol. 1998, 5, 148–155. 10.1038/nsb0298-148. 9461081

[ref75] Lindorff-Larsen K.; Kristjansdottir S.; Teilum K.; Fieber W.; Dobson C. M.; Poulsen F. M.; Vendruscolo M. Determination of an Ensemble of Structures Representing the Denatured State of the Bovine Acyl-Coenzyme a Binding Protein. J. Am. Chem. Soc. 2004, 126, 3291–3299. 10.1021/ja039250g. 15012160

[ref76] Bertoncini C. W.; Jung Y.-S.; Fernandez C. O.; Hoyer W.; Griesinger C.; Jovin T. M.; Zweckstetter M. Release of Long-Range Tertiary Interactions Potentiates Aggregation of Natively Unstructured Alpha-Synuclein. Proc. Natl. Acad. Sci. U.S.A 2005, 102, 1430–1435. 10.1073/pnas.0407146102. 15671169 PMC547830

[ref77] Kristjansdottir S.; Lindorff-Larsen K.; Fieber W.; Dobson C. M.; Vendruscolo M.; Poulsen F. M. Formation of Native and Non-Native Interactions in Ensembles of Denatured ACBP Molecules from Paramagnetic Relaxation Enhancement Studies. J. Mol. Biol. 2005, 347, 1053–1062. 10.1016/j.jmb.2005.01.009. 15784263

[ref78] Felitsky D. J.; Lietzow M. A.; Dyson H. J.; Wright P. E. Modeling Transient Collapsed States of an Unfolded Protein to Provide Insights into Early Folding Events. Proc. Natl. Acad. Sci. U.S.A. 2008, 105, 6278–6283. 10.1073/pnas.0710641105. 18434548 PMC2359776

[ref79] Clore G. M.; Iwahara J. Theory, Practice, and Applications of Paramagnetic Relaxation Enhancement for the Characterization of Transient Low-Population States of Biological Macromolecules and Their Complexes. Chem. Rev. 2009, 109, 4108–4139. 10.1021/cr900033p. 19522502 PMC2825090

[ref80] Feig M.; Brooks C. L. Recent Advances in the Development and Application of Implicit Solvent Models in Biomolecule Simulations. Curr. Opin. Struct. Biol. 2004, 14, 217–224. 10.1016/j.sbi.2004.03.009. 15093837

[ref81] Mackerell A. D. Jr; Feig M.; Brooks C. L. 3rd Extending the Treatment of Backbone Energetics in Protein Force Fields: Limitations of Gas-Phase Quantum Mechanics in Reproducing Protein Conformational Distributions in Molecular Dynamics Simulations. J. Comput. Chem. 2004, 25, 1400–1415. 10.1002/jcc.20065. 15185334

[ref82] Showalter S. A.; Bruschweiler R. Validation of Molecular Dynamics Simulations of Biomolecules Using NMR Spin Relaxation as Benchmarks: Application to the AMBER99SB Force Field. J. Chem. Theory Comput. 2007, 3, 961–975. 10.1021/ct7000045. 26627416

[ref83] Best R. B.; Zhu X.; Shim J.; Lopes P. E. M.; Mittal J.; Feig M.; MacKerell A. D. Optimization of the Additive CHARMM All-Atom Protein Force Field Targeting Improved Sampling of the Backbone Phi, Psi and Side-Chain Chi(1) and Chi(2) Dihedral Angles. J. Chem. Theory Comput. 2012, 8, 3257–3273. 10.1021/ct300400x. 23341755 PMC3549273

[ref84] Lindorff-Larsen K.; Piana S.; Palmo K.; Maragakis P.; Klepeis J. L.; Dror R. O.; shaw D. E. Improved Side-Chain Torsion Potentials for the Amber Ff99SB Protein Force Field. Proteins 2010, 78, 1950–1958. 10.1002/prot.22711. 20408171 PMC2970904

[ref85] Cerutti D. S.; Swope W. C.; Rice J. E.; Case D. A. Ff14ipq: A Self-Consistent Force Field for Condensed-Phase Simulations of Proteins. J. Chem. Theory Comput. 2014, 10, 4515–4534. 10.1021/ct500643c. 25328495 PMC4196740

[ref86] Levy R. M.; Karplus M.; McCammon J. A. Molecular Dynamics Studies of NMR Relaxation in Proteins. Biophys. J. 1980, 32, 628–630. 10.1016/S0006-3495(80)84998-8. 19431400 PMC1327365

[ref87] Bruschweiler R.; Roux B.; Blackledge M.; Griesinger C.; Karplus M.; Ernst R. Influence Of Rapid Intramolecular Motion On Nmr Cross-Relaxation Rates - A Molecular-Dynamics Study Of Antamanide In Solution. J. Am. Chem. Soc. 1992, 114, 2289–2302. 10.1021/ja00033a002.

[ref88] Hornak V.; Abel R.; Okur A.; Strockbine B.; Roitberg A.; Simmerling C. Comparison of Multiple Amber Force Fields and Development of Improved Protein Backbone Parameters. Proteins 2006, 65, 712–725. 10.1002/prot.21123. 16981200 PMC4805110

[ref89] Beauchamp K. A.; Lin Y.-S.; Das R.; Pande V. S. Are Protein Force Fields Getting Better? A Systematic Benchmark on 524 Diverse NMR Measurements. J. Chem. Theory Comput. 2012, 8, 1409–1414. 10.1021/ct2007814. 22754404 PMC3383641

[ref90] Piana S.; Donchev A. G.; Robustelli P.; Shaw D. E. Water Dispersion Interactions Strongly Influence Simulated Structural Properties of Disordered Protein States. J. Phys. Chem. B 2015, 119, 5113–5123. 10.1021/jp508971m. 25764013

[ref91] Zapletal V.; Mládek A.; Melková K.; Louša P.; Nomilner E.; Jasenáková Z.; Kubán V.; Makovická M.; Laníková A.; Žídek L.; et al. Choice of Force Field for Proteins Containing Structured and Intrinsically Disordered Regions. Biophys. J. 2020, 118, 1621–1633. 10.1016/j.bpj.2020.02.019. 32367806 PMC7136338

[ref92] Palmer A. NMR Characterization of the Dynamics of Biomacromolecules. Chem. Rev. 2004, 104, 3623–3640. 10.1021/cr030413t. 15303831

[ref93] Alexandrescu A.; Shortlet D. Backbone Dynamics of a Highly Disordered 131-Residue Fragment of Staphylococcal Nuclease. J. Mol. Biol. 1994, 242, 527–546. 10.1006/jmbi.1994.1598. 7932708

[ref94] Farrow N.; Zhang O.; Formankay J.; Kay L. Comparison of the Backbone Dynamics of a Folded and an Unfolded Sh3 Domain Existing in Equilibrium in Aqueous Buffer. Biochemistry 1995, 34, 868–878. 10.1021/bi00003a021. 7827045

[ref95] Frank M.; Clore G.; Gronenborn A. Structural and Dynamic Characterization of the Urea Denatured State of the Immunoglobulin Binding Domain of Streptococcal Protein-G by Multidimensional Heteronuclear Nmr-Spectroscopy. Protein Sci. 1995, 4, 2605–2615. 10.1002/pro.5560041218. 8580852 PMC2143036

[ref96] Buck M.; Schwalbe H.; Dobson C. M. Main-Chain Dynamics of a Partially Folded Protein: 15N NMR Relaxation Measurements of Hen Egg White Lysozyme Denatured in Trifluoroethanol. J. Mol. Biol. 1996, 257, 669–683. 10.1006/jmbi.1996.0193. 8648632

[ref97] Brutscher B.; Brüschweiler R.; Ernst R. R. Backbone Dynamics and Structural Characterization of the Partially Folded A State of Ubiquitin by 1H, 13C, and 15N Nuclear Magnetic Resonance Spectroscopy. Biochemistry 1997, 36, 13043–13053. 10.1021/bi971538t. 9335566

[ref98] Schwalbe H.; Fiebig K. M.; Buck M.; Jones J. A.; Grimshaw S. B.; Spencer A.; Glaser S. J.; Smith L. J.; Dobson C. M. Structural and Dynamical Properties of a Denatured Protein. Heteronuclear 3D NMR Experiments and Theoretical Simulations of Lysozyme in 8 M Urea. Biochemistry 1997, 36, 8977–8991. 10.1021/bi970049q. 9220986

[ref99] Buevich A. V.; Baum J. Dynamics of Unfolded Proteins: Incorporation of Distributions of Correlation Times in the Model Free Analysis of NMR Relaxation Data. J. Am. Chem. Soc. 1999, 121, 8671–8672. 10.1021/ja9910412.

[ref100] Yang D. W.; Mok Y. K.; Muhandiram D. R.; Forman-Kay J. D.; Kay L. E. H-1-C-13 Dipole-Dipole Cross-Correlated Spin Relaxation as a Probe of Dynamics in Unfolded Proteins: Application to the DrkN SH3 Domain. J. Am. Chem. Soc. 1999, 121, 3555–3556. 10.1021/ja9900914.

[ref101] Tollinger M.; Skrynnikov N. R.; Mulder F. a. A.; Forman-Kay J. D.; Kay L. E. Slow Dynamics in Folded and Unfolded States of an SH3 Domain. J. Am. Chem. Soc. 2001, 123, 11341–11352. 10.1021/ja011300z. 11707108

[ref102] Yao J.; Chung J.; Eliezer D.; Wright P. E.; Dyson H. J. NMR Structural and Dynamic Characterization of the Acid-Unfolded State of Apomyoglobin Provides Insights into the Early Events in Protein Folding. Biochemistry 2001, 40, 3561–3571. 10.1021/bi002776i. 11297422

[ref103] Ochsenbein F.; Neumann J. M.; Guittet E.; Van Heijenoort C. Dynamical Characterization of Residual and Non-Native Structures in a Partially Folded Protein by N-15 NMR Relaxation Using a Model Based on a Distribution of Correlation Times. Protein Sci. 2002, 11, 957–964. 10.1110/ps.4000102. 11910038 PMC2373535

[ref104] Klein-Seetharaman J.; Oikawa M.; Grimshaw S. B.; Wirmer J.; Duchardt E.; Ueda T.; Imoto T.; Smith L. J.; Dobson C. M.; Schwalbe H. Long-Range Interactions within a Nonnative Protein. Science 2002, 295, 1719–1722. 10.1126/science.1067680. 11872841

[ref105] Choy W. Y.; Shortle D.; Kay L. E. Side Chain Dynamics in Unfolded Protein States: An NMR Based H-2 Spin Relaxation Study of Delta 131 Delta. J. Am. Chem. Soc. 2003, 125, 1748–1758. 10.1021/ja021179b. 12580600

[ref106] Wirmer J.; Peti W.; Schwalbe H. Motional Properties of Unfolded Ubiquitin: A Model for a Random Coil Protein. J. Biomol. NMR 2006, 35, 175–186. 10.1007/s10858-006-9026-9. 16865418

[ref107] Le Duff C. S.; Whittaker S. B.-M.; Radford S. E.; Moore G. R. Characterisation of the Conformational Properties of Urea-Unfolded Im7: Implications for the Early Stages of Protein Folding. J. Mol. Biol. 2006, 364, 824–835. 10.1016/j.jmb.2006.09.037. 17045607

[ref108] Houben K.; Blanchard L.; Blackledge M.; Marion D. Intrinsic Dynamics of the Partly Unstructured PX Domain from the Sendai Virus RNA Polymerase Cofactor P. Biophys. J. 2007, 93, 2830–2844. 10.1529/biophysj.107.108829. 17586564 PMC1989709

[ref109] Ebert M.-O.; Bae S.-H.; Dyson H. J.; Wright P. E. NMR Relaxation Study of the Complex Formed between CBP and the Activation Domain of the Nuclear Hormone Receptor Coactivator ACTR. Biochemistry 2008, 47, 1299–1308. 10.1021/bi701767j. 18177052

[ref110] Modig K.; Poulsen F. M. Model-Independent Interpretation of NMR Relaxation Data for Unfolded Proteins: The Acid-Denatured State of ACBP. J. Biomol. NMR 2008, 42, 163–177. 10.1007/s10858-008-9280-0. 18850278

[ref111] Silvers R.; Sziegat F.; Tachibana H.; Segawa S.; Whittaker S.; Günther U. L.; Gabel F.; Huang J.; Blackledge M.; Wirmer-Bartoschek J.; et al. Modulation of Structure and Dynamics by Disulfide Bond Formation in Unfolded States. J. Am. Chem. Soc. 2012, 134, 6846–6854. 10.1021/ja3009506. 22414027

[ref112] Sziegat F.; Silvers R.; Hähnke M.; Jensen M. R.; Blackledge M.; Wirmer-Bartoschek J.; Schwalbe H. Disentangling the Coil: Modulation of Conformational and Dynamic Properties by Site-Directed Mutation in the Non-Native State of Hen Egg White Lysozyme. Biochemistry 2012, 51, 3361–3372. 10.1021/bi300222f. 22468860

[ref113] Konrat R. NMR Contributions to Structural Dynamics Studies of Intrinsically Disordered Proteins. J. Magn. Reson. 2014, 241, 74–85. 10.1016/j.jmr.2013.11.011. 24656082 PMC3985426

[ref114] Kurzbach D.; Schwarz T. C.; Platzer G.; Hoefler S.; Hinderberger D.; Konrat R. Compensatory Adaptations of Structural Dynamics in an Intrinsically Disordered Protein Complex. Angew. Chem., Int. Ed. 2014, 53, 3840–3843. 10.1002/anie.201308389.

[ref115] Prompers J. J.; Bruschweiler R. General Framework for Studying the Dynamics of Folded and Nonfolded Proteins by NMR Relaxation Spectroscopy and MD Simulation. J. Am. Chem. Soc. 2002, 124, 4522–4534. 10.1021/ja012750u. 11960483

[ref116] Xue Y.; Skrynnikov N. R. Motion of a Disordered Polypeptide Chain as Studied by Paramagnetic Relaxation Enhancements, 15N Relaxation, and Molecular Dynamics Simulations: How Fast Is Segmental Diffusion in Denatured Ubiquitin?. J. Am. Chem. Soc. 2011, 133, 14614–14628. 10.1021/ja201605c. 21819149

[ref117] Lindorff-Larsen K.; Trbovic N.; Maragakis P.; Piana S.; Shaw D. E. Structure and Dynamics of an Unfolded Protein Examined by Molecular Dynamics Simulation. J. Am. Chem. Soc. 2012, 134, 3787–3791. 10.1021/ja209931w. 22339051

[ref118] Robustelli P.; Trbovic N.; Friesner R. A.; Palmer A. G. Conformational Dynamics of the Partially Disordered Yeast Transcription Factor GCN4. J. Chem. Theory Comput. 2013, 9, 5190–5200. 10.1021/ct400654r.

[ref119] Markwick P. R. L.; Bouvignies G.; Salmon L.; McCammon J. A.; Nilges M.; Blackledge M. Toward a Unified Representation of Protein Structural Dynamics in Solution. J. Am. Chem. Soc. 2009, 131, 16968–16975. 10.1021/ja907476w. 19919148 PMC2779067

[ref120] Rauscher S.; Gapsys V.; Gajda M. J.; Zweckstetter M.; de Groot B. L.; Grubmüller H. Structural Ensembles of Intrinsically Disordered Proteins Depend Strongly on Force Field: A Comparison to Experiment. J. Chem. Theory Comput. 2015, 11, 5513–5524. 10.1021/acs.jctc.5b00736. 26574339

[ref121] Pietrek L. M.; Stelzl L. S.; Hummer G. Hierarchical Ensembles of Intrinsically Disordered Proteins at Atomic Resolution in Molecular Dynamics Simulations. J. Chem. Theory Comput. 2020, 16, 725–737. 10.1021/acs.jctc.9b00809. 31809054

[ref122] Fawzi N. L.; Phillips A. H.; Ruscio J. Z.; Doucleff M.; Wemmer D. E.; Head-Gordon T. Structure and Dynamics of the A Ss(21–30) Peptide from the Interplay of NMR Experiments and Molecular Simulations. J. Am. Chem. Soc. 2008, 130, 6145–6158. 10.1021/ja710366c. 18412346 PMC3474854

[ref123] Shrestha U. R.; Smith J. C.; Petridis L. Full Structural Ensembles of Intrinsically Disordered Proteins from Unbiased Molecular Dynamics Simulation. Commun. Biol. 2021, 4, 243–250. 10.1038/s42003-021-01759-1. 33623120 PMC7902620

[ref124] Best R. B.; Buchete N.-V.; Hummer G. Are Current Molecular Dynamics Force Fields Too Helical?. Biophys. J. 2008, 95, L07–09. 10.1529/biophysj.108.132696. 18456823 PMC2426634

[ref125] Salvi N.; Abyzov A.; Blackledge M. Multi-Timescale Dynamics in Intrinsically Disordered Proteins from NMR Relaxation and Molecular Simulation. J. Phys. Chem. Lett. 2016, 7, 2483–2489. 10.1021/acs.jpclett.6b00885. 27300592

[ref126] Huang J.; Rauscher S.; Nawrocki G.; Ran T.; Feig M.; de Groot B. L.; Grubmüller H.; MacKerell A. D. CHARMM36m: An Improved Force Field for Folded and Intrinsically Disordered Proteins. Nat. Methods 2017, 14, 71–73. 10.1038/nmeth.4067. 27819658 PMC5199616

[ref127] Vitalis A.; Pappu R. V. ABSINTH: A New Continuum Solvation Model for Simulations of Polypeptides in Aqueous Solutions. J. Comput. Chem. 2009, 30, 673–699. 10.1002/jcc.21005. 18506808 PMC2670230

[ref128] Best R. B.; Hummer G. Optimized Molecular Dynamics Force Fields Applied to the Helix-Coil Transition of Polypeptides. J. Phys. Chem. B 2009, 113, 9004–9015. 10.1021/jp901540t. 19514729 PMC3115786

[ref129] Mercadante D.; Milles S.; Fuertes G.; Svergun D. I.; Lemke E. A.; Gräter F. Kirkwood-Buff Approach Rescues Overcollapse of a Disordered Protein in Canonical Protein Force Fields. J. Phys. Chem. B 2015, 119, 7975–7984. 10.1021/acs.jpcb.5b03440. 26030189

[ref130] Ye W.; Ji D.; Wang W.; Luo R.; Chen H.-F. Test and Evaluation of Ff99IDPs Force Field for Intrinsically Disordered Proteins. J. Chem. Inf. Model. 2015, 55, 1021–1029. 10.1021/acs.jcim.5b00043. 25919886 PMC5490450

[ref131] Carr H. Y.; Purcell E. M. Effects of Diffusion on Free Precession in Nuclear Magnetic Resonance Experiments. Phys. Rev. 1954, 94, 630–638. 10.1103/PhysRev.94.630.

[ref132] Meiboom S.; Gill D. Modified Spin-Echo Method for Measuring Nuclear Relaxation Times. Rev. Sci. Instrum. 1958, 29, 688–691. 10.1063/1.1716296.

[ref133] Forsén S.; Hoffman R. A. Study of Moderately Rapid Chemical Exchange Reactions by Means of Nuclear Magnetic Double Resonance. J. Chem. Phys. 1963, 39, 2892–2901. 10.1063/1.1734121.

[ref134] Kopple K.; Wang Y.; Cheng A.; Bhandary K. Conformations Of Cyclic Octapeptides.5. Crystal-Structure Of Cyclo(Cys-Gly-Pro-Phe)2 And Rotating Frame Relaxation (T1-Rho) Nmr-Studies Of Internal Mobility In Cyclic Octapeptides. J. Am. Chem. Soc. 1988, 110, 4168–4176. 10.1021/ja00221a012.

[ref135] Palmer A. G.; Massi F. Characterization of the Dynamics of Biomacromolecules Using Rotating-Frame Spin Relaxation NMR Spectroscopy. Chem. Rev. 2006, 106, 1700–1719. 10.1021/cr0404287. 16683750

[ref136] Baldwin A. J.; Kay L. E. NMR Spectroscopy Brings Invisible Protein States into Focus. Nat. Chem. Biol. 2009, 5, 808–814. 10.1038/nchembio.238. 19841630

[ref137] Tompa P.; Schad E.; Tantos A.; Kalmar L. Intrinsically Disordered Proteins: Emerging Interaction Specialists. Curr. Opin. Struct. Biol. 2015, 35, 49–59. 10.1016/j.sbi.2015.08.009. 26402567

[ref138] Papaleo E.; Saladino G.; Lambrughi M.; Lindorff-Larsen K.; Gervasio F. L.; Nussinov R. The Role of Protein Loops and Linkers in Conformational Dynamics and Allostery. Chem. Rev. 2016, 116, 6391–6423. 10.1021/acs.chemrev.5b00623. 26889708

[ref139] Delaforge E.; Milles S.; Huang J.-R.; Bouvier D.; Jensen M. R.; Sattler M.; Hart D. J.; Blackledge M. Investigating the Role of Large-Scale Domain Dynamics in Protein-Protein Interactions. Front. Mol. Biosci. 2016, 3, 54 10.3389/fmolb.2016.00054. 27679800 PMC5020063

[ref140] Das R. K.; Pappu R. V. Conformations of Intrinsically Disordered Proteins Are Influenced by Linear Sequence Distributions of Oppositely Charged Residues. Proc. Natl. Acad. Sci. U.S.A. 2013, 110, 13392–13397. 10.1073/pnas.1304749110. 23901099 PMC3746876

[ref141] Wang J.; Choi J.-M.; Holehouse A. S.; Lee H. O.; Zhang X.; Jahnel M.; Maharana S.; Lemaitre R.; Pozniakovsky A.; Drechsel D.; et al. Molecular Grammar Governing the Driving Forces for Phase Separation of Prion-like RNA Binding Proteins. Cell 2018, 174, 688–699. 10.1016/j.cell.2018.06.006. 29961577 PMC6063760

[ref142] Choi J.-M.; Holehouse A. S.; Pappu R. V. Physical Principles Underlying the Complex Biology of Intracellular Phase Transitions. Annu. Rev. Biophys. 2020, 49, 107–133. 10.1146/annurev-biophys-121219-081629. 32004090 PMC10715172

[ref143] Fawzi N. L.; Parekh S. H.; Mittal J. Biophysical Studies of Phase Separation Integrating Experimental and Computational Methods. Curr. Opin. Struct. Biol. 2021, 70, 78–86. 10.1016/j.sbi.2021.04.004. 34144468 PMC8530909

[ref144] Saar K. L.; Morgunov A. S.; Qi R.; Arter W. E.; Krainer G.; Lee A. A.; Knowles T. P. J. Learning the Molecular Grammar of Protein Condensates from Sequence Determinants and Embeddings. Proc. Natl. Acad. Sci. U.S.A. 2021, 118, e2019053118 10.1073/pnas.2019053118. 33827920 PMC8053968

[ref145] Mittag T.; Orlicky S.; Choy W.-Y.; Tang X.; Lin H.; Sicheri F.; Kay L. E.; Tyers M.; Forman-Kay J. D. Dynamic Equilibrium Engagement of a Polyvalent Ligand with a Single-Site Receptor. Proc. Natl. Acad. Sci. U.S.A 2008, 105, 17772–17777. 10.1073/pnas.0809222105. 19008353 PMC2582940

[ref146] Selenko P.; Frueh D. P.; Elsaesser S. J.; Haas W.; Gygi S. P.; Wagner G. In Situ Observation of Protein Phosphorylation by High-Resolution NMR Spectroscopy. Nat. Struct. Mol. Biol. 2008, 15, 321–329. 10.1038/nsmb.1395. 18297086

[ref147] Theillet F.-X.; Rose H. M.; Liokatis S.; Binolfi A.; Thongwichian R.; Stuiver M.; Selenko P. Site-Specific NMR Mapping and Time-Resolved Monitoring of Serine and Threonine Phosphorylation in Reconstituted Kinase Reactions and Mammalian Cell Extracts. Nat. Protoc. 2013, 8, 1416–1432. 10.1038/nprot.2013.083. 23807285

[ref148] Savastano A.; Flores D.; Kadavath H.; Biernat J.; Mandelkow E.; Zweckstetter M. Disease-Associated Tau Phosphorylation Hinders Tubulin Assembly within Tau Condensates. Ang. Chem., Int. Ed. 2021, 60, 726–730. 10.1002/anie.202011157.

[ref149] Salvi N.; Salmon L.; Blackledge M. Dynamic Descriptions of Highly Flexible Molecules from NMR Dipolar Couplings: Physical Basis and Limitations. J. Am. Chem. Soc. 2017, 139, 5011–5014. 10.1021/jacs.7b01566. 28290683

[ref150] Saupe A.; Englert G. High-Resolution Nuclear Magnetic Resonance Spectra of Orientated Molecules. Phys. Rev. Lett. 1963, 11, 462–464. 10.1103/PhysRevLett.11.462.

[ref151] Bernado P.; Blanchard L.; Timmins P.; Marion D.; Ruigrok R. W. H.; Blackledge M. A Structural Model for Unfolded Proteins from Residual Dipolar Couplings and Small-Angle x-Ray Scattering. Proc. Natl. Acad. Sci. U.S.A. 2005, 102, 17002–17007. 10.1073/pnas.0506202102. 16284250 PMC1287987

[ref152] Ozenne V.; Bauer F.; Salmon L.; Huang J.-R.; Jensen M. R.; Segard S.; Bernadó P.; Charavay C.; Blackledge M. Flexible-Meccano: A Tool for the Generation of Explicit Ensemble Descriptions of Intrinsically Disordered Proteins and Their Associated Experimental Observables. Bioinformatics 2012, 28, 1463–1470. 10.1093/bioinformatics/bts172. 22613562

[ref153] Motácková V.; Sanderová H.; Zídek L.; Novácek J.; Padrta P.; Svenková A.; Korelusová J.; Jonák J.; Krásný L.; Sklenár V. Solution Structure of the N-Terminal Domain of Bacillus Subtilis Delta Subunit of RNA Polymerase and Its Classification Based on Structural Homologs. Proteins 2010, 78, 1807–1810. 10.1002/prot.22708. 20310067

[ref154] Rabatinová A.; Šanderová H.; Jirát Matejčková J.; Korelusová J.; Sojka L.; Barvík I.; Papoušková V.; Sklenár V.; Žídek L.; Krásný L. The δ Subunit of RNA Polymerase Is Required for Rapid Changes in Gene Expression and Competitive Fitness of the Cell. J. Bacteriol. 2013, 195, 2603–2611. 10.1128/JB.00188-13. 23543716 PMC3676059

[ref155] Papouskova V.; Kaderavek P.; Otrusinova O.; Rabatinova A.; Sanderova H.; Novacek J.; Krasny L.; Sklenar V.; Zidek L. Structural Study of the Partially Disordered Full-Length Delta Subunit of RNA Polymerase from Bacillus Subtilis. Chembiochem 2013, 14, 1772–1779. 10.1002/cbic.201300226. 23868186

[ref156] Kubán V.; Srb P.; Štégnerová H.; Padrta P.; Zachrdla M.; Jasenáková Z.; Šanderová H.; Vítovská D.; Krásný L.; Koval’ T.; et al. Quantitative Conformational Analysis of Functionally Important Electrostatic Interactions in the Intrinsically Disordered Region of Delta Subunit of Bacterial RNA Polymerase. J. Am. Chem. Soc. 2019, 141, 16817–16828. 10.1021/jacs.9b07837. 31550880

[ref157] Abragam A. The Principles of Nuclear Magnetism; Clarendon Press, 1994; pp 289–305.

[ref158] Goldman M. Quantum Description of High-Resolution NMR in Liquids; Clarendon Press, 1988; pp 231–250.

[ref159] Peng J. W.; Wagner G. Mapping of the Spectral Densities of N-H Bond Motions in Eglin c Using Heteronuclear Relaxation Experiments. Biochemistry 1992, 31, 8571–8586. 10.1021/bi00151a027. 1390643

[ref160] Peng J. W.; Wagner G. Frequency Spectrum of NH Bonds in Eglin c from Spectral Density Mapping at Multiple Fields. Biochemistry 1995, 34, 16733–16752. 10.1021/bi00051a023. 8527448

[ref161] Farrow N.; Zhang O.; Szabo A.; Torchia D.; Kay L. Spectral Density-Function Mapping Using N-15 Relaxation Data Exclusively. J. Biomol. NMR 1995, 6, 153–162. 10.1007/BF00211779. 8589604

[ref162] Ishima R.; Nagayama K. Protein Backbone Dynamics Revealed by Quasi Spectral Density Function Analysis of Amide N-15 Nuclei. Biochemistry 1995, 34, 3162–3171. 10.1021/bi00010a005. 7880811

[ref163] Ishima R.; Yamasaki K.; Nagayama K. Application of the Quasi-Spectral Density Function of (15)N Nuclei to the Selection of a Motional Model for Model-Free Analysis. J. Biomol. NMR 1995, 6, 423–426. 10.1007/BF00197640. 22910879

[ref164] Kaderavek P.; Zapletal V.; Rabatinova A.; Krasny L.; Sklenar V.; Zidek L. Spectral Density Mapping Protocols for Analysis of Molecular Motions in Disordered Proteins. J. Biomol. NMR 2014, 58, 193–207. 10.1007/s10858-014-9816-4. 24515886

[ref165] Halle B.; Wennerström H. Interpretation of Magnetic Resonance Data from Water Nuclei in Heterogeneous Systems. J. Chem. Phys. 1981, 75, 1928–1943. 10.1063/1.442218.

[ref166] Lipari G.; Szabo A. Model-Free Approach To The Interpretation Of Nuclear Magnetic-Resonance Relaxation In Macromolecules.1. Theory And Range Of Validity. J. Am. Chem. Soc. 1982, 104, 4546–4559. 10.1021/ja00381a009.

[ref167] Lipari G.; Szabo A. Model-Free Approach To The Interpretation Of Nuclear Magnetic-Resonance Relaxation In Macromolecules.2. Analysis Of Experimental Results. J. Am. Chem. Soc. 1982, 104, 4559–4570. 10.1021/ja00381a010.

[ref168] Clore G.; Szabo A.; Bax A.; Kay L.; Driscoll P.; Gronenborn A. Deviations from the Simple 2-Parameter Model-Free Approach to the Interpretation of N-15 Nuclear Magnetic-Relaxation of Proteins. J. Am. Chem. Soc. 1990, 112, 4989–4991. 10.1021/ja00168a070.

[ref169] Halle B. The Physical Basis of Model-Free Analysis of NMR Relaxation Data from Proteins and Complex Fluids. J. Chem. Phys. 2009, 131, 224507 10.1063/1.3269991. 20001057

[ref170] Farrow N. A.; Zhang O. W.; FormanKay J. D.; Kay L. E. Characterization of the Backbone Dynamics of Folded and Denatured States of an SH3 Domain. Biochemistry 1997, 36, 2390–2402. 10.1021/bi962548h. 9054544

[ref171] Yang D.; Kay L. E. Contributions to Conformational Entropy Arising from Bond Vector Fluctuations Measured from NMR-Derived Order Parameters: Application to Protein Folding. J. Mol. Biol. 1996, 263, 369–382. 10.1006/jmbi.1996.0581. 8913313

[ref172] Yang D.; Mok Y.-K.; Forman-Kay J. D.; Farrow N. A.; Kay L. E. Contributions to Protein Entropy and Heat Capacity from Bond Vector Motions Measured by NMR Spin Relaxation1. J. Mol. Biol. 1997, 272, 790–804. 10.1006/jmbi.1997.1285. 9368658

[ref173] Ochsenbein F.; Guerois R.; Neumann J.-M.; Sanson A.; Guittet E.; van Heijenoort C. 15N NMR Relaxation as a Probe for Helical Intrinsic Propensity: The Case of the Unfolded D2 Domain of Annexin I. J. Biomol NMR 2001, 19, 3–18. 10.1023/A:1008390606077. 11246852

[ref174] Cole K. S.; Cole R. H. Dispersion and Absorption in Dielectrics I. Alternating Current Characteristics. J. Chem. Phys. 1941, 9, 341–351. 10.1063/1.1750906.

[ref175] Bovey F. A.; Mirau P. A. NMR of Polymers; Academic Press, 1996; pp 361–376.

[ref176] Cho M.-K.; Kim H.-Y.; Bernado P.; Fernandez C. O.; Blackledge M.; Zweckstetter M. Amino Acid Bulkiness Defines the Local Conformations and Dynamics of Natively Unfolded Alpha-Synuclein and Tau. J. Am. Chem. Soc. 2007, 129, 3032 10.1021/ja067482k. 17315997

[ref177] Parigi G.; Rezaei-Ghaleh N.; Giachetti A.; Becker S.; Fernandez C.; Blackledge M.; Griesinger C.; Zweckstetter M.; Luchinat C. Long-Range Correlated Dynamics in Intrinsically Disordered Proteins. J. Am. Chem. Soc. 2014, 136, 16201–16209. 10.1021/ja506820r. 25331250

[ref178] Bae S.-H.; Dyson H. J.; Wright P. E. Prediction of the Rotational Tumbling Time for Proteins with Disordered Segments. J. Am. Chem. Soc. 2009, 131, 6814–6821. 10.1021/ja809687r. 19391622 PMC2694746

[ref179] Walsh J. D.; Meier K.; Ishima R.; Gronenborn A. M. NMR Studies on Domain Diffusion and Alignment in Modular GB1 Repeats. Biophys. J. 2010, 99, 2636–2646. 10.1016/j.bpj.2010.08.036. 20959105 PMC2955504

[ref180] Amoros D.; Ortega A.; Garcia de la Torre J. Prediction of Hydrodynamic and Other Solution Properties of Partially Disordered Proteins with a Simple, Coarse-Grained Model. J. Chem. Theory Comput. 2013, 9, 1678–1685. 10.1021/ct300948u. 26587628

[ref181] Rezaei-Ghaleh N.; Klama F.; Munari F.; Zweckstetter M. Predicting the Rotational Tumbling of Dynamic Multidomain Proteins and Supramolecular Complexes. Angew. Chem., Int. Ed. Engl. 2013, 52, 11410–11414. 10.1002/anie.201305094. 24000220

[ref182] Sugase K.; Dyson H. J.; Wright P. E. Mechanism of Coupled Folding and Binding of an Intrinsically Disordered Protein. Nature 2007, 447, 1021-U11 10.1038/nature05858. 17522630

[ref183] Hilser V. J.; Thompson E. B. Intrinsic Disorder as a Mechanism to Optimize Allosteric Coupling in Proteins. Proc. Natl. Acad. Sci. U. S. A. 2007, 104, 8311–8315. 10.1073/pnas.0700329104. 17494761 PMC1895946

[ref184] Kiefhaber T.; Bachmann A.; Jensen K. S. Dynamics and Mechanisms of Coupled Protein Folding and Binding Reactions. Curr. Opin. Struct. Biol. 2012, 22, 21–29. 10.1016/j.sbi.2011.09.010. 22129832

[ref185] Marsh J. A.; Teichmann S. A.; Forman-Kay J. D. Probing the Diverse Landscape of Protein Flexibility and Binding. Curr. Opin. Struct. Biol. 2012, 22, 643–650. 10.1016/j.sbi.2012.08.008. 22999889

[ref186] Rogers J. M.; Steward A.; Clarke J. Folding and Binding of an Intrinsically Disordered Protein: Fast, but Not “Diffusion-Limited.. J. Am. Chem. Soc. 2013, 135, 1415–1422. 10.1021/ja309527h. 23301700 PMC3776562

[ref187] Iesmantavicius V.; Dogan J.; Jemth P.; Teilum K.; Kjaergaard M. Helical Propensity in an Intrinsically Disordered Protein Accelerates Ligand Binding. Angew. Chem., Int. Ed. 2014, 53, 1548–1551. 10.1002/anie.201307712.

[ref188] Rogers J. M.; Wong C. T.; Clarke J. Coupled Folding and Binding of the Disordered Protein PUMA Does Not Require Particular Residual Structure. J. Am. Chem. Soc. 2014, 136, 5197–5200. 10.1021/ja4125065. 24654952 PMC4017604

[ref189] Rogers J. M.; Oleinikovas V.; Shammas S. L.; Wong C. T.; De Sancho D.; Baker C. M.; Clarke J. Interplay between Partner and Ligand Facilitates the Folding and Binding of an Intrinsically Disordered Protein. Proc. Natl. Acad. Sci. U.S.A. 2014, 111, 15420–15425. 10.1073/pnas.1409122111. 25313042 PMC4217413

[ref190] Flock T.; Weatheritt R. J.; Latysheva N. S.; Babu M. M. Controlling Entropy to Tune the Functions of Intrinsically Disordered Regions. Curr. Opin. Struct. Biol. 2014, 26, 62–72. 10.1016/j.sbi.2014.05.007. 24930020

[ref191] Schneider R.; Maurin D.; Communie G.; Kragelj J.; Hansen D. F.; Ruigrok R. W. H.; Jensen M. R.; Blackledge M. Visualizing the Molecular Recognition Trajectory of an Intrinsically Disordered Protein Using Multinuclear Relaxation Dispersion NMR. J. Am. Chem. Soc. 2015, 137, 1220–1229. 10.1021/ja511066q. 25551399

[ref192] Gianni S.; Dogan J.; Jemth P. Coupled Binding and Folding of Intrinsically Disordered Proteins: What Can We Learn from Kinetics?. Curr. Opin. Struct. Biol. 2016, 36, 18–24. 10.1016/j.sbi.2015.11.012. 26720267

[ref193] Khan S. N.; Charlier C.; Augustyniak R.; Salvi N.; Déjean V.; Bodenhausen G.; Lequin O.; Pelupessy P.; Ferrage F. Distribution of Pico- and Nanosecond Motions in Disordered Proteins from Nuclear Spin Relaxation. Biophys. J. 2015, 109, 988–999. 10.1016/j.bpj.2015.06.069. 26331256 PMC4564687

[ref194] Gill M. L.; Byrd R. A.; Palmer A. G. III Dynamics of GCN4 Facilitate DNA Interaction: A Model-Free Analysis of an Intrinsically Disordered Region. Phys. Chem. Chem. Phys. 2016, 18, 5839–5849. 10.1039/C5CP06197K. 26661739 PMC4894059

[ref195] Brady J. P.; Farber P. J.; Sekhar A.; Lin Y.-H.; Huang R.; Bah A.; Nott T. J.; Chan H. S.; Baldwin A. J.; Forman-Kay J. D.; et al. Structural and Hydrodynamic Properties of an Intrinsically Disordered Region of a Germ Cell-Specific Protein on Phase Separation. Proc. Natl. Acad. Sci. U.S.A. 2017, 114, E8194–E8203. 10.1073/pnas.1706197114. 28894006 PMC5625912

[ref196] Ryan V. H.; Dignon G. L.; Zerze G. H.; Chabata C. V.; Silva R.; Conicella A. E.; Amaya J.; Burke K. A.; Mittal J.; Fawzi N. L. Mechanistic View of HnRNPA2 Low-Complexity Domain Structure, Interactions, and Phase Separation Altered by Mutation and Arginine Methylation. Mol. Cell 2018, 69, 465–479. 10.1016/j.molcel.2017.12.022. 29358076 PMC5801700

[ref197] Murthy A. C.; Dignon G. L.; Kan Y.; Zerze G. H.; Parekh S. H.; Mittal J.; Fawzi N. L. Molecular Interactions Underlying Liquid–liquid Phase Separation of the FUS Low-Complexity Domain. Nat. Struct. Mol. Biol. 2019, 26, 637–648. 10.1038/s41594-019-0250-x. 31270472 PMC6613800

[ref198] Theillet F.-X.; Binolfi A.; Bekei B.; Martorana A.; Rose H. M.; Stuiver M.; Verzini S.; Lorenz D.; van Rossum M.; Goldfarb D.; Selenko P. Structural Disorder of Monomeric α-Synuclein Persists in Mammalian Cells. Nature 2016, 530, 45–50. 10.1038/nature16531. 26808899

[ref199] Abyzov A.; Salvi N.; Schneider R.; Maurin D.; Ruigrok R. W. H.; Jensen M. R.; Blackledge M. Identification of Dynamic Modes in an Intrinsically Disordered Protein Using Temperature-Dependent NMR Relaxation. J. Am. Chem. Soc. 2016, 138, 6240–6251. 10.1021/jacs.6b02424. 27112095

[ref200] Lewandowski J. R.; Halse M. E.; Blackledge M.; Emsley L. Protein Dynamics. Direct Observation of Hierarchical Protein Dynamics. Science 2015, 348, 578–581. 10.1126/science.aaa6111. 25931561

[ref201] Kimmich R.; Anoardo E. Field-Cycling NMR Relaxometry. Prog. Nucl. Magn. Reson. Spectrosc. 2004, 44, 257–320. 10.1016/j.pnmrs.2004.03.002.

[ref202] Bryant R. G.; Korb J. P. Nuclear Magnetic Resonance and Spin Relaxation in Biological Systems. Magn. Reson. Imaging 2005, 23, 167–173. 10.1016/j.mri.2004.11.026. 15833608

[ref203] Rouse P. J. A theory of the linear viscoelastic properties of dilute solutions of coiling polymers. J. Chem. Phys. 1953, 21, 1272 10.1063/1.1699180.

[ref204] Young W. S.; Brooks C. L. III A Microscopic View of Helix Propagation: N and C-Terminal Helix Growth in Alanine Helices. J. Mol. Biol. 1996, 259, 560–572. 10.1006/jmbi.1996.0339. 8676388

[ref205] Mikhonin A. V.; Asher S. A. Direct UV Raman Monitoring of 310-Helix and π-Bulge Premelting during α-Helix Unfolding. J. Am. Chem. Soc. 2006, 128, 13789–13795. 10.1021/ja062269+. 17044707

[ref206] Best R. B.; Mittal J. Balance between α and β Structures in Ab Initio Protein Folding. J. Phys. Chem. B 2010, 114, 8790–8798. 10.1021/jp102575b. 20536262

[ref207] Adamski W.; Salvi N.; Maurin D.; Magnat J.; Milles S.; Jensen M. R.; Abyzov A.; Moreau C. J.; Blackledge M. A Unified Description of Intrinsically Disordered Protein Dynamics under Physiological Conditions Using NMR Spectroscopy. J. Am. Chem. Soc. 2019, 141, 17817–17829. 10.1021/jacs.9b09002. 31591893

[ref208] Theillet F.-X.; Binolfi A.; Frembgen-Kesner T.; Hingorani K.; Sarkar M.; Kyne C.; Li C.; Crowley P. B.; Gierasch L.; Pielak G. J.; et al. Physicochemical Properties of Cells and Their Effects on Intrinsically Disordered Proteins (IDPs). Chem. Rev. 2014, 114, 6661–6714. 10.1021/cr400695p. 24901537 PMC4095937

[ref209] Gruebele M.; Pielak G. J. Dynamical Spectroscopy and Microscopy of Proteins in Cells. Curr. Opin. Struct. Biol. 2021, 70, 1–7. 10.1016/j.sbi.2021.02.001. 33662744

[ref210] Selenko P.; Serber Z.; Gadea B.; Ruderman J.; Wagner G. Quantitative NMR Analysis of the Protein G B1 Domain in Xenopus Laevis Egg Extracts and Intact Oocytes. Proc. Natl. Acad. Sci. U.S.A. 2006, 103, 11904–11909. 10.1073/pnas.0604667103. 16873549 PMC1523310

[ref211] Li C.; Charlton L. M.; Lakkavaram A.; Seagle C.; Wang G.; Young G. B.; Macdonald J. M.; Pielak G. J. Differential Dynamical Effects of Macromolecular Crowding on an Intrinsically Disordered Protein and a Globular Protein: Implications for in-Cell NMR Spectroscopy. J. Am. Chem. Soc. 2008, 130, 6310–6311. 10.1021/ja801020z. 18419123 PMC2435198

[ref212] Ito Y.; Selenko P. Cellular Structural Biology. Curr. Opin. Struct. Biol. 2010, 20, 640–648. 10.1016/j.sbi.2010.07.006. 20801639

[ref213] Smith A. E.; Zhang Z.; Pielak G. J.; Li C. NMR Studies of Protein Folding and Binding in Cells and Cell-like Environments. Curr. Opin. Struct. Biol. 2015, 30, 7–16. 10.1016/j.sbi.2014.10.004. 25479354

[ref214] Majumder S.; Xue J.; DeMott C. M.; Reverdatto S.; Burz D. S.; Shekhtman A. Probing Protein Quinary Interactions by In-Cell Nuclear Magnetic Resonance Spectroscopy. Biochemistry 2015, 54, 2727–2738. 10.1021/acs.biochem.5b00036. 25894651 PMC4447238

[ref215] Luchinat E.; Banci L. In-Cell NMR in Human Cells: Direct Protein Expression Allows Structural Studies of Protein Folding and Maturation. Acc. Chem. Res. 2018, 51, 1550–1557. 10.1021/acs.accounts.8b00147. 29869502

[ref216] Guseman A. J.; Perez Goncalves G. M.; Speer S. L.; Young G. B.; Pielak G. J. Protein Shape Modulates Crowding Effects. Proc. Natl. Acad. Sci. U.S.A. 2018, 115, 10965–10970. 10.1073/pnas.1810054115. 30301792 PMC6205421

[ref217] Zimmerman S. B.; Trach S. O. Estimation of Macromolecule Concentrations and Excluded Volume Effects for the Cytoplasm of Escherichia Coli. J. Mol. Biol. 1991, 222, 599–620. 10.1016/0022-2836(91)90499-V. 1748995

[ref218] Ellis R. J. Macromolecular Crowding: Obvious but Underappreciated. Trends Biochem. Sci. 2001, 26, 597–604. 10.1016/S0968-0004(01)01938-7. 11590012

[ref219] Zeskind B. J.; Jordan C. D.; Timp W.; Trapani L.; Waller G.; Horodincu V.; Ehrlich D. J.; Matsudaira P. Nucleic Acid and Protein Mass Mapping by Live-Cell Deep-Ultraviolet Microscopy. Nat. Methods 2007, 4, 567–569. 10.1038/nmeth1053. 17546037

[ref220] Cohen R. D.; Pielak G. J. A Cell Is More than the Sum of Its (Dilute) Parts: A Brief History of Quinary Structure. Protein Sci. 2017, 26, 403–413. 10.1002/pro.3092. 27977883 PMC5326556

[ref221] Davey N. E. The Functional Importance of Structure in Unstructured Protein Regions. Curr. Opin. Struct. Biol. 2019, 56, 155–163. 10.1016/j.sbi.2019.03.009. 31003202

[ref222] von Bülow S.; Siggel M.; Linke M.; Hummer G. Dynamic Cluster Formation Determines Viscosity and Diffusion in Dense Protein Solutions. Proc. Natl. Acad. Sci. U.S.A. 2019, 116, 9843–9852. 10.1073/pnas.1817564116. 31036655 PMC6525548

[ref223] Wei M.-T.; Elbaum-Garfinkle S.; Holehouse A. S.; Chen C. C.-H.; Feric M.; Arnold C. B.; Priestley R. D.; Pappu R. V.; Brangwynne C. P. Phase Behaviour of Disordered Proteins Underlying Low Density and High Permeability of Liquid Organelles. Nat. Chem. 2017, 9, 1118–1125. 10.1038/nchem.2803. 29064502 PMC9719604

[ref224] Brangwynne C. P.; Eckmann C. R.; Courson D. S.; Rybarska A.; Hoege C.; Gharakhani J.; Jülicher F.; Hyman A. A. Germline P Granules Are Liquid Droplets That Localize by Controlled Dissolution/Condensation. Science 2009, 324, 1729–1732. 10.1126/science.1172046. 19460965

[ref225] Alberti S.; Gladfelter A.; Mittag T. Considerations and Challenges in Studying Liquid-Liquid Phase Separation and Biomolecular Condensates. Cell 2019, 176, 419–434. 10.1016/j.cell.2018.12.035. 30682370 PMC6445271

[ref226] Schuler B.; Eaton W. A. Protein Folding Studied by Single-Molecule FRET. Curr. Opin. Struct. Biol. 2008, 18, 16–26. 10.1016/j.sbi.2007.12.003. 18221865 PMC2323684

[ref227] Echeverria I.; Makarov D. E.; Papoian G. A. Concerted Dihedral Rotations Give Rise to Internal Friction in Unfolded Proteins. J. Am. Chem. Soc. 2014, 136, 8708–8713. 10.1021/ja503069k. 24844314

[ref228] König I.; Zarrine-Afsar A.; Aznauryan M.; Soranno A.; Wunderlich B.; Dingfelder F.; Stüber J. C.; Plückthun A.; Nettels D.; Schuler B. Single-Molecule Spectroscopy of Protein Conformational Dynamics in Live Eukaryotic Cells. Nat. Methods 2015, 12, 773–779. 10.1038/nmeth.3475. 26147918

[ref229] König I.; Soranno A.; Nettels D.; Schuler B. Impact of In-Cell and In-Vitro Crowding on the Conformations and Dynamics of an Intrinsically Disordered Protein. Ang. Chem., Int. Ed. 2021, 60, 10724–10729. 10.1002/anie.202016804.

[ref230] Paudel B. P.; Fiorini E.; Börner R.; Sigel R. K. O.; Rueda D. S. Optimal Molecular Crowding Accelerates Group II Intron Folding and Maximizes Catalysis. Proc. Natl. Acad. Sci. U.S.A. 2018, 115, 11917–11922. 10.1073/pnas.1806685115. 30397128 PMC6255197

[ref231] McConkey E. H. Molecular Evolution, Intracellular Organization, and the Quinary Structure of Proteins. Proc. Natl. Acad. Sci. U.S.A. 1982, 79, 3236–3240. 10.1073/pnas.79.10.3236. 6954476 PMC346390

[ref232] Monteith W. B.; Cohen R. D.; Smith A. E.; Guzman-Cisneros E.; Pielak G. J. Quinary Structure Modulates Protein Stability in Cells. Proc. Natl. Acad. Sci. U.S.A. 2015, 112, 1739–1742. 10.1073/pnas.1417415112. 25624496 PMC4330749

[ref233] Danielsson J.; Mu X.; Lang L.; Wang H.; Binolfi A.; Theillet F.-X.; Bekei B.; Logan D. T.; Selenko P.; Wennerström H.; et al. Thermodynamics of Protein Destabilization in Live Cells. Proc. Natl. Acad. Sci. U.S.A. 2015, 112, 12402–12407. 10.1073/pnas.1511308112. 26392565 PMC4603463

[ref234] Song X.; Lv T.; Chen J.; Wang J.; Yao L. Characterization of Residue Specific Protein Folding and Unfolding Dynamics in Cells. J. Am. Chem. Soc. 2019, 141, 11363–11366. 10.1021/jacs.9b04435. 31305080

[ref235] Sakai T.; Tochio H.; Tenno T.; Ito Y.; Kokubo T.; Hiroaki H.; Shirakawa M. In-Cell NMR Spectroscopy of Proteins inside Xenopus Laevis Oocytes. J. Biomol. NMR 2006, 36, 179–188. 10.1007/s10858-006-9079-9. 17031531

[ref236] Bodart J.-F.; Wieruszeski J.-M.; Amniai L.; Leroy A.; Landrieu I.; Rousseau-Lescuyer A.; Vilain J.-P.; Lippens G. NMR Observation of Tau in Xenopus Oocytes. J. Magn. Reson. 2008, 192, 252–257. 10.1016/j.jmr.2008.03.006. 18378475

[ref237] Wang Q.; Zhuravleva A.; Gierasch L. M. Exploring Weak, Transient Protein–Protein Interactions in Crowded in Vivo Environments by in-Cell Nuclear Magnetic Resonance Spectroscopy. Biochemistry 2011, 50, 9225–9236. 10.1021/bi201287e. 21942871 PMC3202675

[ref238] Waudby C. A.; Mantle M. D.; Cabrita L. D.; Gladden L. F.; Dobson C. M.; Christodoulou J. Rapid Distinction of Intracellular and Extracellular Proteins Using NMR Diffusion Measurements. J. Am. Chem. Soc. 2012, 134, 11312–11315. 10.1021/ja304912c. 22694283

[ref239] Dedmon M. M.; Patel C. N.; Young G. B.; Pielak G. J. FlgM Gains Structure in Living Cells. Proc. Natl. Acad. Sci. U.S.A. 2002, 99, 12681–12684. 10.1073/pnas.202331299. 12271132 PMC130520

[ref240] Ye Y.; Liu X.; Zhang Z.; Wu Q.; Jiang B.; Jiang L.; Zhang X.; Liu M.; Pielak G. J.; Li C. 19F NMR Spectroscopy as a Probe of Cytoplasmic Viscosity and Weak Protein Interactions in Living Cells. Chem. Eur. J. 2013, 19, 12705–12710. 10.1002/chem.201301657. 23922149

[ref241] Sekhar A.; Latham M. P.; Vallurupalli P.; Kay L. E. Viscosity-Dependent Kinetics of Protein Conformational Exchange: Microviscosity Effects and the Need for a Small Viscogen. J. Phys. Chem. B 2014, 118, 4546–4551. 10.1021/jp501583t. 24707961

[ref242] Roos M.; Ott M.; Hofmann M.; Link S.; Rössler E.; Balbach J.; Krushelnitsky A.; Saalwächter K. Coupling and Decoupling of Rotational and Translational Diffusion of Proteins under Crowding Conditions. J. Am. Chem. Soc. 2016, 138, 10365–10372. 10.1021/jacs.6b06615. 27434647

[ref243] Bai J.; Liu M.; Pielak G. J.; Li C. Macromolecular and Small Molecular Crowding Have Similar Effects on α-Synuclein Structure. ChemPhysChem 2017, 18, 55–58. 10.1002/cphc.201601097. 27860069

[ref244] Leeb S.; Yang F.; Oliveberg M.; Danielsson J. Connecting Longitudinal and Transverse Relaxation Rates in Live-Cell NMR. J. Phys. Chem. B 2020, 124, 10698–10707. 10.1021/acs.jpcb.0c08274. 33179918 PMC7735724

[ref245] Persson E.; Halle B. Cell Water Dynamics on Multiple Time Scales. Proc. Natl. Acad. Sci. U.S.A. 2008, 105, 6266–6271. 10.1073/pnas.0709585105. 18436650 PMC2359779

[ref246] Kimmich R.; Fatkullin N. Self-Diffusion Studies by Intra- and Inter-Molecular Spin-Lattice Relaxometry Using Field-Cycling: Liquids, Plastic Crystals, Porous Media, and Polymer Segments. Prog. Nucl. Magn. Reson. Spectrosc. 2017, 101, 18–50. 10.1016/j.pnmrs.2017.04.001. 28844220

[ref247] Korb J.-P. Multiscale Nuclear Magnetic Relaxation Dispersion of Complex Liquids in Bulk and Confinement. Prog. Nucl. Magn. Reson. Spectrosc. 2018, 104, 12–55. 10.1016/j.pnmrs.2017.11.001. 29405980

[ref248] Cukier R. I. Diffusion of Brownian Spheres in Semidilute Polymer Solutions. Macromolecules 1984, 17, 252–255. 10.1021/ma00132a023.

[ref249] Barshtein G.; Almagor A.; Yedgar S.; Gavish B. Inhomogeneity of Viscous Aqueous-Solutions. Phys. Rev. E 1995, 52, 555–557. 10.1103/PhysRevE.52.555.

[ref250] Lavalette D.; Tétreau C.; Tourbez M.; Blouquit Y. Microscopic Viscosity and Rotational Diffusion of Proteins in a Macromolecular Environment. Biophys. J. 1999, 76, 2744–2751. 10.1016/S0006-3495(99)77427-8. 10233089 PMC1300244

[ref251] Szymański J.; Patkowski A.; Wilk A.; Garstecki P.; Holyst R. Diffusion and Viscosity in a Crowded Environment: From Nano- to Macroscale. J. Phys. Chem. B 2006, 110, 25593–25597. 10.1021/jp0666784. 17181192

[ref252] Sekhar A.; Vallurupalli P.; Kay L. E. Defining a Length Scale for Millisecond-Timescale Protein Conformational Exchange. Proc. Natl. Acad. Sci. U.S.A. 2013, 110, 11391–11396. 10.1073/pnas.1303273110. 23801755 PMC3710843

[ref253] Kalwarczyk T.; Sozanski K.; Ochab-Marcinek A.; Szymanski J.; Tabaka M.; Hou S.; Holyst R. Motion of Nanoprobes in Complex Liquids within the Framework of the Length-Scale Dependent Viscosity Model. Adv. Colloid Interface Sci. 2015, 223, 55–63. 10.1016/j.cis.2015.06.007. 26189602

[ref254] Wisniewska A.; Sozanski K.; Kalwarczyk T.; Kedra-Krolik K.; Holyst R. Scaling Equation for Viscosity of Polymer Mixtures in Solutions with Application to Diffusion of Molecular Probes. Macromolecules 2017, 50, 4555–4561. 10.1021/acs.macromol.7b00545.

[ref255] Qin S.; Zhou H.-X. Protein Folding, Binding, and Droplet Formation in Cell-like Conditions. Curr. Opin. Struct. Biol. 2017, 43, 28–37. 10.1016/j.sbi.2016.10.006. 27771543 PMC5397379

[ref256] Luh L. M.; Hänsel R.; Löhr F.; Kirchner D. K.; Krauskopf K.; Pitzius S.; Schäfer B.; Tufar P.; Corbeski I.; Güntert P.; et al. Molecular Crowding Drives Active Pin1 into Nonspecific Complexes with Endogenous Proteins Prior to Substrate Recognition. J. Am. Chem. Soc. 2013, 135, 13796–13803. 10.1021/ja405244v. 23968199

[ref257] Salvi N.; Abyzov A.; Blackledge M. Analytical Description of NMR Relaxation Highlights Correlated Dynamics in Intrinsically Disordered Proteins. Angew. Chem.. Int. Ed. 2017, 56, 14020–14024. 10.1002/anie.201706740.

[ref258] Abascal J. L. F.; Vega C. A General Purpose Model for the Condensed Phases of Water: TIP4P/2005. J. Chem. Phys. 2005, 123, 234505 10.1063/1.2121687. 16392929

[ref259] Chandrasekhar I.; Clore G.; Szabo A.; Gronenborn A.; Brooks B. A 500-Ps Molecular-Dynamics Simulation Study of Interleukin-1-Beta in Water - Correlation with Nuclear-Magnetic-Resonance Spectroscopy and Crystallography. J. Mol. Biol. 1992, 226, 239–250. 10.1016/0022-2836(92)90136-8. 1619653

[ref260] Salmon L.; Pierce L.; Grimm A.; Roldan J.-L. O.; Mollica L.; Jensen M. R.; van Nuland N.; Markwick P. R. L.; McCammon J. A.; Blackledge M. Multi-Timescale Conformational Dynamics of the SH3 Domain of CD2-Associated Protein Using NMR Spectroscopy and Accelerated Molecular Dynamics. Angew. Chem., Int. Ed. 2012, 51, 6103–6106. 10.1002/anie.201202026.

[ref261] Salvi N.; Abyzov A.; Blackledge M. Solvent-Dependent Segmental Dynamics in Intrinsically Disordered Proteins. Sci. Adv. 2019, 5, eaax2348 10.1126/sciadv.aax2348. 31259246 PMC6598773

[ref262] Fung H. Y. J.; Birol M.; Rhoades E. IDPs in macromolecular complexes: the roles of multivalent interactions in diverse assemblies. Curr. Opin. Str. Biol. 2018, 49, 36–43. 10.1016/j.sbi.2017.12.007.

[ref263] Ivarsson Y.; Jemth P. Affinity and Specificity of Motif-Based Protein-Protein Interactions. Curr. Opin. Struct. Biol. 2019, 54, 26–33. 10.1016/j.sbi.2018.09.009. 30368054

[ref264] Milles S.; Salvi N.; Blackledge M.; Jensen M. R. Characterization of Intrinsically Disordered Proteins and Their Dynamic Complexes: From in Vitro to Cell-like Environments. Prog. Nucl. Magn. Reson. Spectrosc. 2018, 109, 79–100. 10.1016/j.pnmrs.2018.07.001. 30527137

[ref265] Bugge K.; Brakti I.; Fernandes C. B.; Dreier J. E.; Lundsgaard J. E.; Olsen J. G.; Skriver K.; Kragelund B. B. Interactions by Disorder - A Matter of Context. Front. Mol. Biosci. 2020, 7, 110 10.3389/fmolb.2020.00110. 32613009 PMC7308724

[ref266] Dyson H. J.; Wright P. E. NMR Illuminates Intrinsic Disorder. Curr. Opin. Struct. Biol. 2021, 70, 44–52. 10.1016/j.sbi.2021.03.015. 33951592 PMC8530845

[ref267] Schneider R.; Blackledge M.; Jensen M. R. Elucidating Binding Mechanisms and Dynamics of Intrinsically Disordered Protein Complexes Using NMR Spectroscopy. Curr. Opin. Struct. Biol. 2019, 54, 10–18. 10.1016/j.sbi.2018.09.007. 30316104

[ref268] Xie M.; Hansen A. L.; Yuan J.; Brüschweiler R. Residue-Specific Interactions of an Intrinsically Disordered Protein with Silica Nanoparticles and Their Quantitative Prediction. J. Phys. Chem. C Nanomater Interfaces 2016, 120, 24463–24468. 10.1021/acs.jpcc.6b08213. 28337243 PMC5358802

[ref269] Xie M.; Li D.-W.; Yuan J.; Hansen A. L.; Brüschweiler R. Quantitative Binding Behavior of Intrinsically Disordered Proteins to Nanoparticle Surfaces at Individual Residue Level. Chemistry 2018, 24, 16997–17001. 10.1002/chem.201804556. 30240067

[ref270] Xie M.; Yu L.; Bruschweiler-Li L.; Xiang X.; Hansen A. L.; Brüschweiler R. Functional Protein Dynamics on Uncharted Time Scales Detected by Nanoparticle-Assisted NMR Spin Relaxation. Sci. Adv. 2019, 5, eaax5560 10.1126/sciadv.aax5560. 31453342 PMC6693908

[ref271] Vallurupalli P.; Bouvignies G.; Kay L. E. Studying “Invisible” Excited Protein States in Slow Exchange with a Major State Conformation. J. Am. Chem. Soc. 2012, 134, 8148–8161. 10.1021/ja3001419. 22554188

[ref272] Fawzi N. L.; Ying J.; Ghirlando R.; Torchia D. A.; Clore G. M. Atomic-Resolution Dynamics on the Surface of Amyloid-β Protofibrils Probed by Solution NMR. Nature 2011, 480, 268–272. 10.1038/nature10577. 22037310 PMC3237923

[ref273] Montelione G. T.; Wagner G. 2D Chemical Exchange NMR Spectroscopy by Proton-Detected Heteronuclear Correlation. J. Am. Chem. Soc. 1989, 111, 3096–3098. 10.1021/ja00190a072.

[ref274] Wider G.; Neri D.; Wüthrich K. Studies of Slow Conformational Equilibria in Macromolecules by Exchange of Heteronuclear Longitudinal 2-Spin-Order in a 2D Difference Correlation Experiment. J. Biomol. NMR 1991, 1, 93–98. 10.1007/BF01874572.

[ref275] Jensen M. R.; Houben K.; Lescop E.; Blanchard L.; Ruigrok R. W. H.; Blackledge M. Quantitative Conformational Analysis of Partially Folded Proteins from Residual Dipolar Couplings: Application to the Molecular Recognition Element of Sendai Virus Nucleoprotein. J. Am. Chem. Soc. 2008, 130, 8055–8061. 10.1021/ja801332d. 18507376

[ref276] Fonin A. V.; Darling A. L.; Kuznetsova I. M.; Turoverov K. K.; Uversky V. N. Intrinsically Disordered Proteins in Crowded Milieu: When Chaos Prevails within the Cellular Gumbo. Cell. Mol. Life Sci. 2018, 75, 3907–3929. 10.1007/s00018-018-2894-9. 30066087 PMC11105604

[ref277] Breindel L.; Burz D. S.; Shekhtman A. Interaction Proteomics by Using In-Cell NMR Spectroscopy. J. Proteomics 2019, 191, 202–211. 10.1016/j.jprot.2018.02.006. 29427760 PMC6082733

[ref278] Zosel F.; Soranno A.; Buholzer K. J.; Nettels D.; Schuler B. Depletion Interactions Modulate the Binding between Disordered Proteins in Crowded Environments. Proc. Natl. Acad. Sci. U.S.A. 2020, 117, 13480–13489. 10.1073/pnas.1921617117. 32487732 PMC7306994

[ref279] Kim Y. C.; Bhattacharya A.; Mittal J. Macromolecular Crowding Effects on Coupled Folding and Binding. J. Phys. Chem. B 2014, 118, 12621–12629. 10.1021/jp508046y. 25302571

[ref280] Maldonado A. Y.; Burz D. S.; Reverdatto S.; Shekhtman A. Fate of Pup inside the Mycobacterium Proteasome Studied by In-Cell NMR. PLoS One 2013, 8, e74576 10.1371/journal.pone.0074576. 24040288 PMC3769308

[ref281] Binolfi A.; Limatola A.; Verzini S.; Kosten J.; Theillet F.-X.; Rose H. M.; Bekei B.; Stuiver M.; van Rossum M.; Selenko P. Intracellular Repair of Oxidation-Damaged α-Synuclein Fails to Target C-Terminal Modification Sites. Nat. Commun. 2016, 7, 10251 10.1038/ncomms10251. 26807843 PMC4737712

[ref282] Zhang S.; Wang C.; Lu J.; Ma X.; Liu Z.; Li D.; Liu Z.; Liu C. In-Cell NMR Study of Tau and MARK2 Phosphorylated Tau. Int. J. Mol. Sci. 2019, 20, e90 10.3390/ijms20010090.

[ref283] Yuwen T.; Brady J. P.; Kay L. E. Probing Conformational Exchange in Weakly Interacting, Slowly Exchanging Protein Systems via Off-Resonance R1ρ Experiments: Application to Studies of Protein Phase Separation. J. Am. Chem. Soc. 2018, 140, 2115–2126. 10.1021/jacs.7b09576. 29303268

[ref284] Hough L. E.; Dutta K.; Sparks S.; Temel D. B.; Kamal A.; Tetenbaum-Novatt J.; Rout M. P.; Cowburn D. The Molecular Mechanism of Nuclear Transport Revealed by Atomic-Scale Measurements. Elife 2015, 4, e10027 10.7554/eLife.10027. 26371551 PMC4621360

[ref285] Raveh B.; Karp J. M.; Sparks S.; Dutta K.; Rout M. P.; Sali A.; Cowburn D. Slide-and-Exchange Mechanism for Rapid and Selective Transport through the Nuclear Pore Complex. Proc. Natl. Acad. Sci. U.S.A. 2016, 113, e2489 10.1073/pnas.1522663113. 27091992 PMC4983827

[ref286] Milles S.; Jensen M. R.; Lazert C.; Guseva S.; Ivashchenko S.; Communie G.; Maurin D.; Gerlier D.; Ruigrok R. W. H.; Blackledge M. An Ultraweak Interaction in the Intrinsically Disordered Replication Machinery Is Essential for Measles Virus Function. Sci. Adv. 2018, 4, eaat7778 10.1126/sciadv.aat7778. 30140745 PMC6105297

[ref287] Guseva S.; Milles S.; Jensen M. R.; Schoehn G.; Ruigrok R. W.; Blackledge M. Structure, Dynamics and Phase Separation of Measles Virus RNA Replication Machinery. Curr. Opin. Virol. 2020, 41, 59–67. 10.1016/j.coviro.2020.05.006. 32570195

[ref288] Longhi S.; Bloyet L.-M.; Gianni S.; Gerlier D. How Order and Disorder within Paramyxoviral Nucleoproteins and Phosphoproteins Orchestrate the Molecular Interplay of Transcription and Replication. Cell. Mol. Life Sci. 2017, 74, 3091–3118. 10.1007/s00018-017-2556-3. 28600653 PMC11107670

[ref289] Chang C.; Hou M.-H.; Chang C.-F.; Hsiao C.-D.; Huang T. The SARS Coronavirus Nucleocapsid Protein–Forms and Functions. Antiviral Res. 2014, 103, 39–50. 10.1016/j.antiviral.2013.12.009. 24418573 PMC7113676

[ref290] Savastano A.; Ibáñez de Opakua A.; Rankovic M.; Zweckstetter M. Nucleocapsid Protein of SARS-CoV-2 Phase Separates into RNA-Rich Polymerase-Containing Condensates. Nat. Commun. 2020, 11, 6041 10.1038/s41467-020-19843-1. 33247108 PMC7699647

[ref291] Guseva S.; Perez L. M.; Camacho-Zarco A.; Bessa L. M.; Salvi N.; Malki A.; Maurin D.; Blackledge M. 1H, 13C and 15N Backbone Chemical Shift Assignments of the n-Terminal and Central Intrinsically Disordered Domains of SARS-CoV-2 Nucleoprotein. Biomol. NMR Assign. 2021, 15, 255–260. 10.1007/s12104-021-10014-x. 33730325 PMC7967780

[ref292] Schiavina M.; Pontoriero L.; Uversky V. N.; Felli I. C.; Pierattelli R. The Highly Flexible Disordered Regions of the SARS-CoV-2 Nucleocapsid N Protein within the 1–248 Residue Construct: Sequence-Specific Resonance Assignments through NMR. Biomol. NMR Assign. 2021, 15, 219–227. 10.1007/s12104-021-10009-8. 33660218 PMC7928198

[ref293] Cubuk J.; Alston J. J.; Incicco J. J.; Singh S.; Stuchell-Brereton M. D.; Ward M. D.; Zimmerman M. I.; Vithani N.; Griffith D.; Wagoner J. A.; et al. The SARS-CoV-2 Nucleocapsid Protein Is Dynamic, Disordered, and Phase Separates with RNA. Nat. Commun. 2021, 12, 1936 10.1038/s41467-021-21953-3. 33782395 PMC8007728

[ref294] Bessa L. M.; Guseva S.; Camacho-Zarco A. R.; Salvi N.; Maurin D.; Perez L. M.; Botova M.; Malki A.; Nanao M.; Jensen M. R. The Intrinsically Disordered SARS-CoV-2 Nucleoprotein in Dynamic Complex with Its Viral Partner Nsp3a. Sci. Adv. 2022, 8, eabm4034 10.1126/sciadv.abm4034. 35044811 PMC8769549

[ref295] Delaforge E.; Milles S.; Bouvignies G.; Bouvier D.; Boivin S.; Salvi N.; Maurin D.; Martel A.; Round A.; Lemke E. A.; et al. Large-Scale Conformational Dynamics Control H5N1 Influenza Polymerase PB2 Binding to Importin α. J. Am. Chem. Soc. 2015, 137, 15122–15134. 10.1021/jacs.5b07765. 26424125

[ref296] Boivin S.; Cusack S.; Ruigrok R. W. H.; Hart D. J. Influenza A Virus Polymerase: Structural Insights into Replication and Host Adaptation Mechanisms. J. Biol. Chem. 2010, 285, 28411–28417. 10.1074/jbc.R110.117531. 20538599 PMC2937865

[ref297] Long J. S.; Giotis E. S.; Moncorgé O.; Frise R.; Mistry B.; James J.; Morisson M.; Iqbal M.; Vignal A.; Skinner M. A.; Barclay W. S. Species Difference in ANP32A Underlies Influenza A Virus Polymerase Host Restriction. Nature 2016, 529, 101–104. 10.1038/nature16474. 26738596 PMC4710677

[ref298] Camacho-Zarco A. R.; Kalayil S.; Maurin D.; Salvi N.; Delaforge E.; Milles S.; Jensen M. R.; Hart D. J.; Cusack S.; Blackledge M. Molecular Basis of Host-Adaptation Interactions between Influenza Virus Polymerase PB2 Subunit and ANP32A. Nat. Commun. 2020, 11, 3656 10.1038/s41467-020-17407-x. 32694517 PMC7374565

[ref299] Borgia A.; Borgia M. B.; Bugge K.; Kissling V. M.; Heidarsson P. O.; Fernandes C. B.; Sottini A.; Soranno A.; Buholzer K. J.; Nettels D.; et al. Extreme Disorder in an Ultrahigh-Affinity Protein Complex. Nature 2018, 555, 61–66. 10.1038/nature25762. 29466338 PMC6264893

[ref300] Sottini A.; Borgia A.; Borgia M. B.; Bugge K.; Nettels D.; Chowdhury A.; Heidarsson P. O.; Zosel F.; Best R. B.; Kragelund B. B.; et al. Polyelectrolyte Interactions Enable Rapid Association and Dissociation in High-Affinity Disordered Protein Complexes. Nat. Commun. 2020, 11, 5736 10.1038/s41467-020-18859-x. 33184256 PMC7661507

[ref301] Kragelj J.; Palencia A.; Nanao M. H.; Maurin D.; Bouvignies G.; Blackledge M.; Jensen M. R. Structure and Dynamics of the MKK7-JNK Signaling Complex. Proc. Natl. Acad. Sci. U.S.A. 2015, 112, 3409–3414. 10.1073/pnas.1419528112. 25737554 PMC4371970

[ref302] Delaforge E.; Kragelj J.; Tengo L.; Palencia A.; Milles S.; Bouvignies G.; Salvi N.; Blackledge M.; Jensen M. R. Deciphering the Dynamic Interaction Profile of an Intrinsically Disordered Protein by NMR Exchange Spectroscopy. J. Am. Chem. Soc. 2018, 140, 1148–1158. 10.1021/jacs.7b12407. 29276882

[ref303] Kragelj J.; Orand T.; Delaforge E.; Tengo L.; Blackledge M.; Palencia A.; Jensen M. R. Enthalpy-Entropy Compensation in the Promiscuous Interaction of an Intrinsically Disordered Protein with Homologous Protein Partners. Biomolecules 2021, 11, 1204 10.3390/biom11081204. 34439869 PMC8391806

[ref304] Charlier C.; Bouvignies G.; Pelupessy P.; Walrant A.; Marquant R.; Kozlov M.; De Ioannes P.; Bolik-Coulon N.; Sagan S.; Cortes P.; et al. Structure and Dynamics of an Intrinsically Disordered Protein Region That Partially Folds upon Binding by Chemical-Exchange NMR. J. Am. Chem. Soc. 2017, 139, 12219–12227. 10.1021/jacs.7b05823. 28780862

